# Dynamic interactions within the host-associated microbiota cause tumor formation in the basal metazoan *Hydra*

**DOI:** 10.1371/journal.ppat.1008375

**Published:** 2020-03-19

**Authors:** Kai Rathje, Benedikt Mortzfeld, Marc P. Hoeppner, Jan Taubenheim, Thomas C. G. Bosch, Alexander Klimovich

**Affiliations:** 1 Zoological Institute, Kiel University, Kiel, Germany; 2 Department of Biology, University of Massachusetts Dartmouth, Dartmouth, Massachusetts, United States of America; 3 Institute of Clinical Molecular Biology, Kiel University, Kiel, Germany; 4 Institute for Zoology and Organismic Interactions, Heinrich-Heine University Düsseldorf, Düsseldorf, Germany; Florida State University, UNITED STATES

## Abstract

The extent to which disturbances in the resident microbiota can compromise an animal’s health is poorly understood. *Hydra* is one of the evolutionary oldest animals with naturally occurring tumors. Here, we found a causal relationship between an environmental spirochete (*Turneriella* spec.) and tumorigenesis in *Hydra*. Unexpectedly, virulence of this pathogen requires the presence of *Pseudomonas* spec., a member of *Hydra*´s beneficial microbiome indicating that dynamic interactions between a resident bacterium and a pathogen cause tumor formation. The observation points to the crucial role of commensal bacteria in maintaining tissue homeostasis and adds support to the view that microbial community interactions are essential for disease. These findings in an organism that shares deep evolutionary connections with all animals have implications for our understanding of cancer.

## Author summary

Here we follow up on our initial observation of tumor formation in the basal metazoan *Hydra* and demonstrate that tumor development in one of the evolutionary oldest animals is caused by a dynamic interplay between an environmental spirochete, the host-associated resident microbiota, and the tissue homeostasis within the animal. Unexpectedly, the pathogenicity of the environmental bacterium *Turneriella* is context-dependent: the virulence of this pathogen requires the presence of a member of *Hydra*’s beneficial microbiome—the *Pseudomonas* bacterium. Dynamic interactions between two microbiota members have profound effects onto the host tissue homeostasis and fitness. Our data provide direct evidence for the important role of the resident microbiome in maintaining tissue homeostasis and pathogen defense, a fundamental process that is likely to take place in every tissue of every animal species. In summary, our study uncovers an evolutionary conserved role of the resident microbiome in guarding host’s tissue homeostasis.

## Introduction

Recent studies provided evidence for the deep evolutionary origin of tumor formation [1,2] and the emergence of tumor-related genes at the base of multicellularity [3]. From the beginning of evolution, animals were multiorganismal associations of a multicellular host and microbial community[4]. Microbial symbionts influence virtually all aspects of eukaryote biology [4,5], and their impact on host fitness ranges from detrimental to beneficial, occasionally shifting along this continuum [6]. There is evidence that the presence of certain commensal microbes may turn harmful to the host and cause intoxication [7,8] and inflammation [9]; that some bacteria species including *Helicobacter pylori* [10,11] and *Bacteroides fragilis* [12] may promote tumor formation in the host; and that normally harmless commensal bacteria can become pathogenic under stress conditions [9,13]. A previous study has indicated [14] that the natural microbiota provides resistance against mutagen-induced tumorigenesis in mice. Despite the importance of a resident microbiome, the impact of its individual members on tumor development has yet to be rigorously tested.

The early emerging metazoan *Hydra* is colonized by a stable microbiome [15] and is one of the evolutionary oldest animals with naturally occurring tumors [1] characterized by a differentiation arrest and uncontrolled accumulation of female germline precursor cells. Earlier studies in *Hydra* largely focused on the innate immune system and identified a crucial role of the transcription factor FoxO [16,17] and antimicrobial peptides [15] for maintaining a specific microbiome. Previous work also suggested that interactions among commensal bacteria are essential to prevent pathogen infection [18] underlining the importance of microbiota diversity as a protective factor against disease. However, nothing is known in *Hydra* about the role of the resident microbiome in tumor formation. We have discovered that dynamic interactions between a resident bacterium *Pseudomonas* and an environmental spirochete microbe *Turneriella* induce tumorigenesis and gravely affect tissue homeostasis and fitness of the host. The findings provide evidence that bacteria-driven tumorigenesis has deep evolutionary roots.

## Results

### Presence of a spirochete correlates with tumor formation

**Fig 1:**
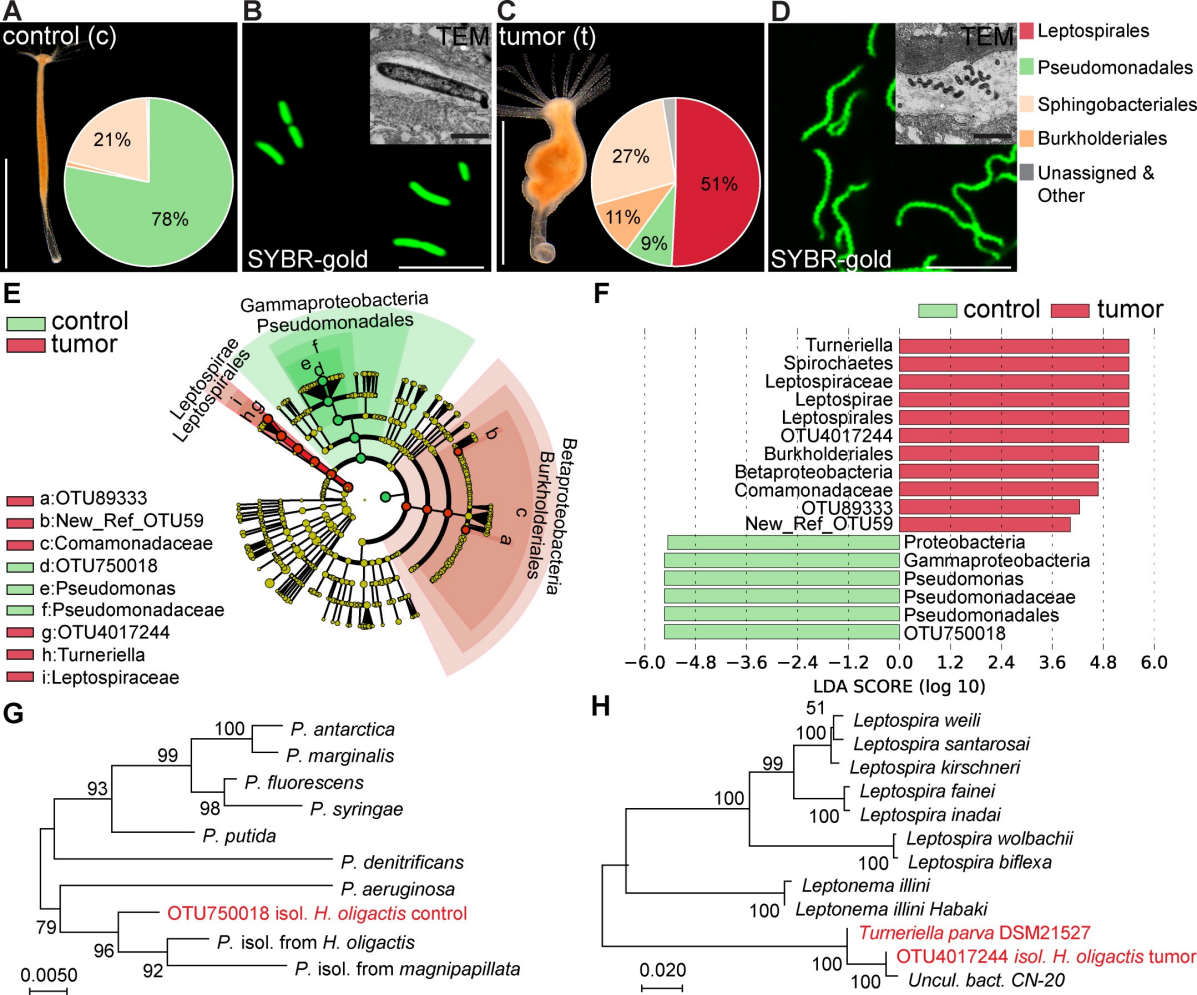
Presence of spirochetes in microbiota of *H*. *oligactis* correlates with tumor formation. **(A)** Phenotype of healthy *H*. *oligactis* polyps is defined by a tube-like body column and a specific microbiome dominated by Pseudomonadales; scale bar: 0.5 cm. Inset: Relative abundance of bacteria identified by 16S rDNA sequencing and resolved at the phylum level, *n* = 6. **(B)** Rod-shaped *Pseudomonas* bacteria colonize the ECM of *Hydra*. Bacteria are visualized on isolated mesoglea with SYBR-gold staining (scale bar: 5 μm) and on TEM-sections (inset, scale bar: 0.5 μm). **(C)** Tumor-bearing polyps of *H*. *oligactis* are characterized by tissue bulges and greatly altered microbiome with dominated by Leptospirales (scale bar: 0.5 cm). Inset: Relative abundance of bacteria identified by 16S rDNA sequencing and presented on the phylum level, *n* = 6. **(D)** Helical bacteria colonize the ECM of tumor-bearing *H*. *oligactis*, visualized on isolated mesoglea with SYBR-gold staining (scale bar: 5 μm) and on TEM-sections (inset, scale bar: 0.5 μm). **(E)** Taxonomic cladogram presenting OTUs differentially represented in the microbiota of control and tumor polyps generated using LEfSe analysis of 16S rDNA sequencing data; red—taxa enriched in tumor polyps; green–taxa enriched in healthy polyps. **(F)** The enrichment of certain taxa in tumor polyps is statistically supported by high positive LDA score values (red), and the taxa strongly enriched in control polyps are supported by negative score values (green); *n* = 6 **(G)** Phylogenetic tree of *Pseudomonas* species clusters the isolate OTU750018 from healthy *H*. *oligactis* together with other Hydra-associated *Pseudomonas* isolates close to the *P*. *aeruginosa* species. Neighbour-joining phylogram with numbers at nodes representing bootstrap support values calculated by 1000 iterations. **(H)** Phylogenetic tree of Leptospiraceae family clusters the spirochetes OTU4017244 isolated from tumor-bearing *H*. *oligactis* with an unculturable clone CN-20 and the reference *T*. *parva* strain DSM21527. For sequence accession numbers see S1 Table. Neighbour-joining phylogram with numbers at nodes representing bootstrap support values calculated by 1000 iterations.

In previous studies we have shown that *Hydra* polyps are colonized by a specific microbiota [15,19] and that tissue homeostasis and microbiota composition may depend on each other [17,20]. We therefore hypothesized that the previously characterized bona fide tumor formation in *Hydra oligactis* [1] could be accompanied by an altered microbiome. To test this hypothesis, we compared the microbiome of tumorous and healthy polyps (further referred to as control) by 16S rDNA sequencing, confocal and electron microscopy. Our analysis revealed that the microbiota of control polyps was dominated by a single bacterium (OTU750018) that can be assigned to the *Pseudomonas* genus (Fig 1A). These findings were supported by microscopic analysis, where a highly abundant rod-shaped bacterium was consistently detected in the mesoglea (extracellular matrix) of healthy polyps (Fig 1B, S1A Fig). In tumor bearing polyps, the abundance of *Pseudomonas* (OTU750018) was greatly reduced (Fig 1C) and the rod-shaped microbes were found only rarely. Instead, the microbiota of tumor bearing polyps was enriched with a *Turneriella* bacterium (OTU4017244) belonging to the spirochete phylum (Fig 1C). Consistent with this, numerous helically coiled bacteria were detected in the mesoglea (Fig 1D, S1B Fig). These findings were strongly supported by a LEfSe [21] analysis which uses the relative abundance of microbes to discover biomarkers that explain the differences between two or more microbial communities (see Methods for details). The *Pseudomonas* OTU750018 and its higher rank taxonomic categories up to the Proteobacteria phylum were under-represented in the tumorous polyps compared to controls (Fig 1E and 1F), while *Turneriella* OTU4017244 and all the ranks up to spirochetes phylum were strongly enriched in the tumorous polyps (Fig 1E and 1F). Additionally, two microbes belonging to the Comamonadaceae family (represented by OTU89333 and OTU59) were also enriched in the tumor-bearing polyps compared to control, yet with lower statistical support (Fig 1E and 1F). Since we have no microscopic evidence for the presence of Comamonadaceae in the mesoglea, and since other observations suggested that bacteria from this group are only transiently colonizing *Hydra* glycocalyx [18], we disregarded these changes in the further analysis.

Phylogenetic analysis of the 16S rDNA provided further insights into the identity of the bacteria colonizing the mesoglea in *Hydra oligactis* (Fig 1G and 1H). The OTU750018 isolated from control polyps clustered close with the other members of the *Pseudomonas* species earlier isolated from *H*. *oligactis* and *H*. *magnipapillata* (Fig 1G, S1 Table). The closest well-characterized relative of this group is *P*. *aeruginosa*. For simplicity, we refer further to OTU750018 isolated from control *H*. *oligactis* as *Pseudomonas*. We were able to isolate this bacterium in pure culture for further analysis. The spirochete OTU4017244 identified in the tumor-bearing *H*. *oligactis* polyps clustered close to the unculturable bacterium CN-20 (Fig 1H). Not surprisingly, all our efforts to cultivate the OTU4017244 failed. For further molecular and functional analysis we therefore used the most closely related and well-characterized strain *Turneriella parva* DSM21527 [22] (Fig 1H). Taken together, these results revealed a strong correlation between the presence of the spirochete *Turneriella* and the tumor formation in *H*. *oligactis*.

### Spirochetes are necessary for tumorigenesis

**Fig 2:**
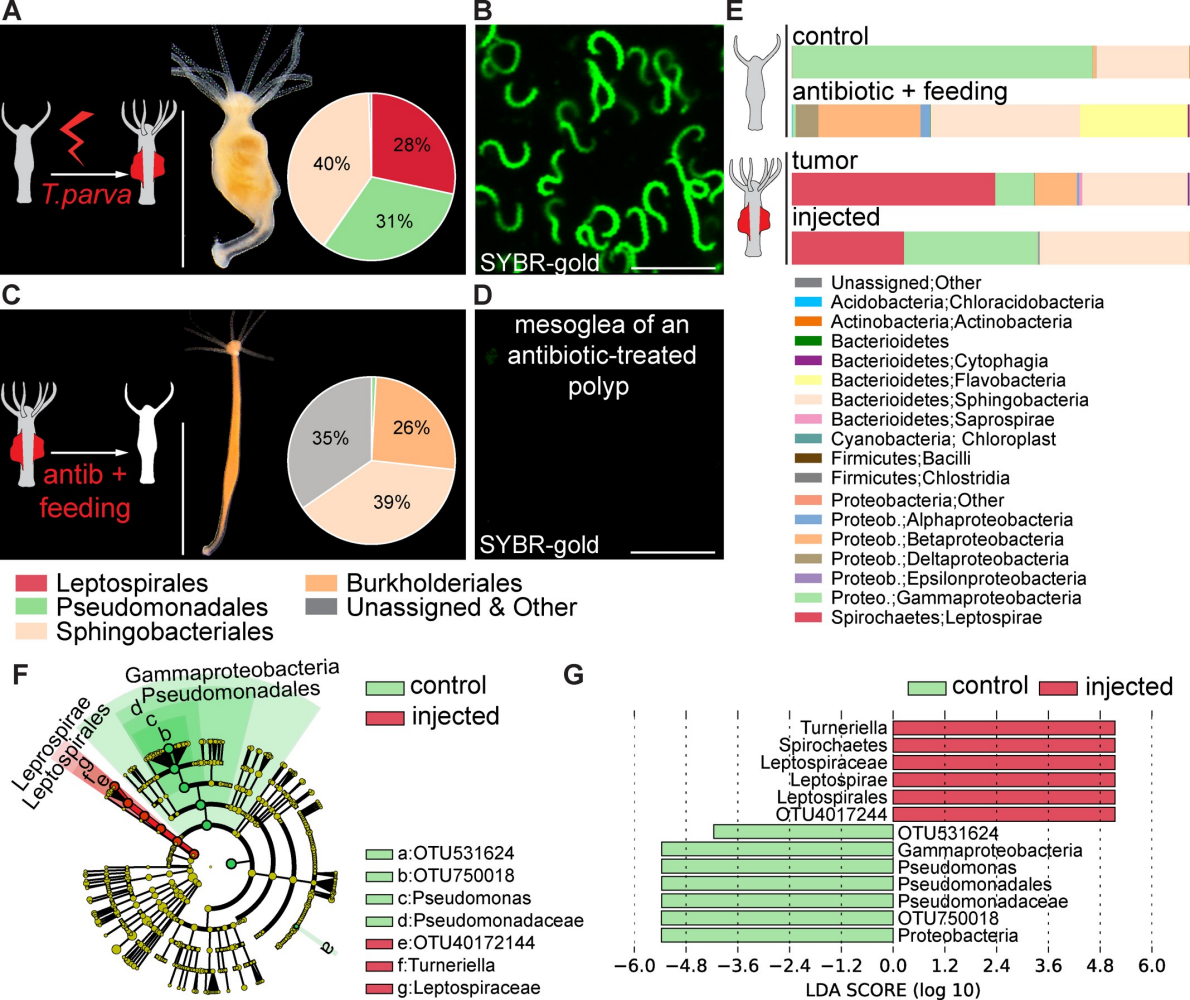
Spirochetes are necessary for tumorigenesis in *H*. *oligactis*. **(A)** Injection of a pure *T*. *parva* culture into healthy polyps (scheme, left) results in acquisition of tumorous phenotype (right) within 4 weeks post injection. Injected polyps show similar tissue outgrowth and Leptospirales-dominated microbiome as natural tumor polyps; scale bar: 0.5 cm. Inset: Relative abundance of bacteria identified by 16S rDNA sequencing and presented on the phylum level, *n* = 6. **(B)** *T*. *parva* densely colonizes the mesoglea of the injected polyps, revealed by SYBR-gold staining (scale bar: 5 μm). **(C)** Removal of the spirochetes from the tumorous polyps by antibiotic treatment (scheme, left) results in recovering of normal phenotype within 2 weeks. The antibiotic-treated polyps (right) show normal body shape and absence of Leptospirales in the microbiome. Inset: Relative abundance of bacteria identified by 16S rDNA sequencing and presented on the phylum level, *n* = 6. **(D)** The mesoglea of antibiotic-treated polyps shows no presence of spirochetes revealed by SYBR-gold staining (scale bar: 5 μm). **(E)** Relative abundance plots of the microbial composition of healthy (control) and antibiotic-treated (antibiotic+feeding) polyps, naturally tumorous polyps (tumor) and tumor-bearing hydras resulted from *T*. *parva* injection (injected) on the bacteria class level. **(F)** Taxonomic cladogram presenting OTUs differentially present in the microbiota of control and *T*. *parva*-injected polyps generated using LEfSe analysis; red—taxa enriched in injected polyps; green–taxa enriched in intact control polyps. **(G)** The enrichment of certain taxa in *T*. *parva*-injected polyps is statistically supported by high positive LDA score values (red), and the taxa strongly enriched in intact control polyps are supported by negative score values (green); *n* = 6.

To test whether there is a causal relationship between spirochetes and tumor formation in *Hydra*, we injected a pure culture of *T*. *parva* into control *H*. *oligactis* polyps (Fig 2A). Four weeks after injection, over 20% of polyps (22 out of 96) had developed tumors (Fig 2A, S2 Fig) which were very similar to the ones observed before (Fig 1C). 16S rDNA analysis and confocal microscopy confirmed that the injected polyps were successfully colonized by the helically coiled *T*. *parva* (Fig 2A and 2B). Notably, the abundance of *Pseudomonas* was greatly reduced in the injected polyps (Fig 2A) suggesting that *T*. *parva* may displace the commensal *Pseudomonas* from its niche in the mesoglea. For the remaining cases (74/96), we could detect neither phenotypic changes nor helically coiled bacteria in the mesoglea nor 16S rDNA sequences corresponding to *T*. *parva* (S3 Fig).

To further determine a functional role of microbes in *H*. *oligactis* tumor formation, we treated the naturally arising tumor-bearing polyps for two weeks with a cocktail of antibiotics (ampicillin, rifampicin, streptomycin, spectinomycin and neomycin, 50 μg/ml each) and thereby eliminated the spirochetes from the mesoglea (Fig 2C and 2D, S4A Fig). Although feeding with *Artemia* nauplii introduced some bacteria into the antibiotics-treated polyps (Fig 2C, S4B Fig), no spirochetes could be detected even weeks later (Fig 2C, 2D and 2E). Enrichment of spirochetes and depletion of *Pseudomonas* in the *T*. *parva* injected polyps was statistically supported by LEfSeq analysis (Fig 2F and 2G). Strikingly, all (55/55) tumor polyps recovered a healthy phenotype within two weeks after antibiotic treatment (Fig 2C, S4 Fig) and phenotypically were indistinguishable from control polyps. In a similar manner, antibiotic treatment of tumorous polyps generated by injection of *T*. *parva* eradicated the tumors (S4C Fig). These results are consistent with the view that colonization with spirochetes is indispensable to elicit tumor formation in *H*. *oligactis*.

### Spirochetes cause developmental alterations and fitness loss

**Fig 3:**
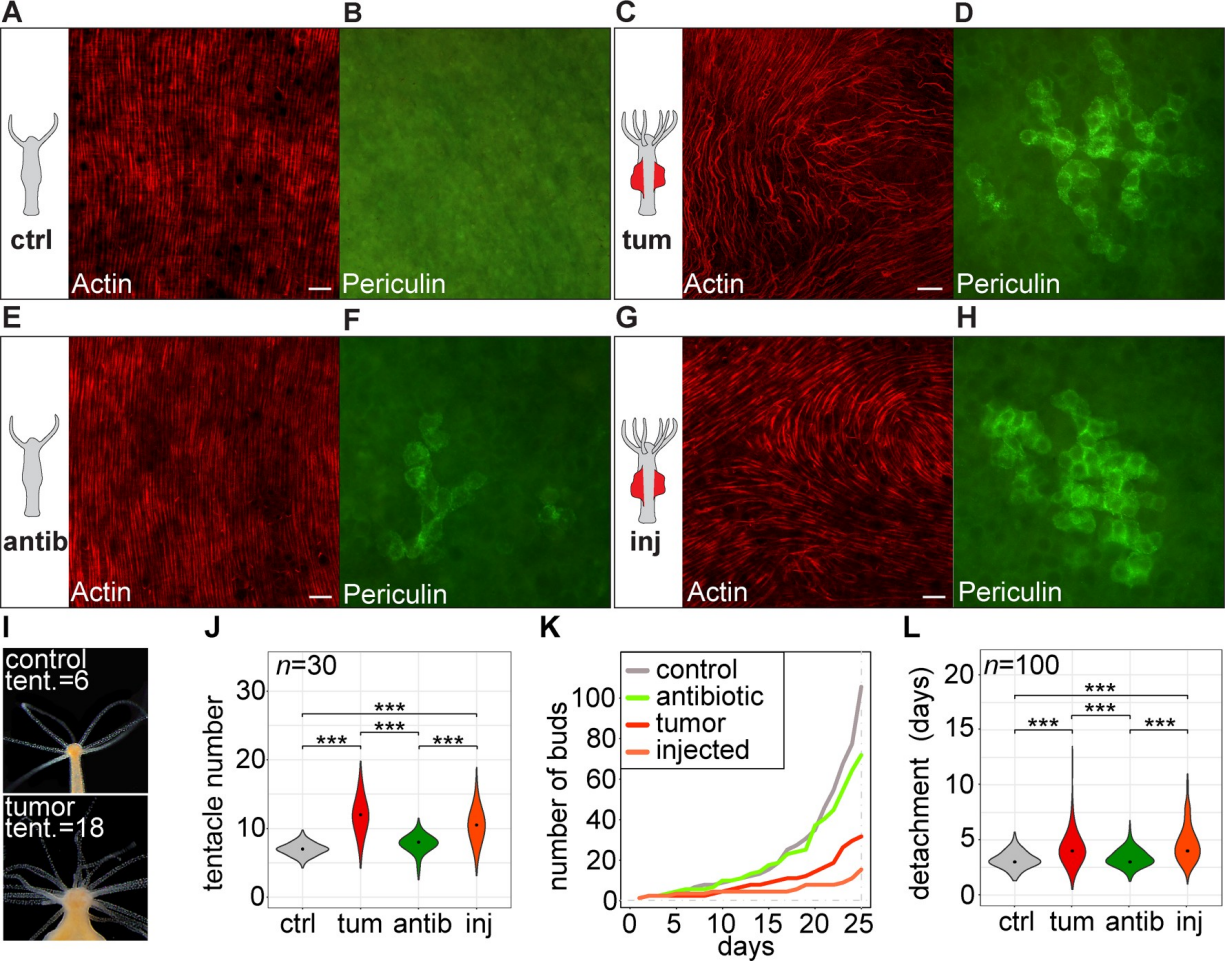
Spirochetes cause developmental alterations and fitness loss in *Hydra*. **(A)** In the control polyps (ctrl), actin fibers in the ectoderm are organized parallel to the polyp body axis, revealed by phalloidin-rhodamine staining, scale bar: 10 μm. **(B)** No germline precursor cells can be detected in control polyps using anti-periculin immunostaining. **(C)** In the naturally-occurring tumorous polyps (tum), actin fibers are disorganized, scale bar: 10 μm. **(D)** Periculin-positive germline precursor cells accumulate in the gastric region of tumor polyps, consistent with the previous observations (Domazet-Loso *et al*., 2014). **(E)** Antibiotics treatment (antib) of tumorous polyps reverts the cytoskeleton structure to normal. **(F)** In antibiotics-treated polyps, the density of periculin-positive germline cells declines. **(G)** In the polyps injected with *T*. *parva* (inj), actin cytoskeleton is disorganized similar to naturally-occurring tumors. **(H)** Numerous periculin-positive germline cells appear after injection of *T*. *parva* into control polyps. **(I)** Tumorous *H*. *oligactis* polyps have higher number of tentacles compared to control animals. **(J)** Tentacle number is significantly higher in tumorous polyps (tum) compared to controls (ctrl). Antibiotics treatment reduces the number back to the normal level, while *T*. *parva* injection increases the tentacle number almost to the tumor polyp level. ***—p<0.001, *—p<0.05. **(K)** Population growth rate measured as number of buds generated in 25 days is reduced in naturally tumorous polyps and *T*. *parva*-injected polyps compared to the healthy controls and antibiotic-treated polyps. **(L)** Bud detachment time is increased in the tumorous polyps (tum) compared to healthy controls (ctrl). Antibiotics treatment of tumorous polyps (antib) brings detachment time to the normal level, and *T*. *parva* injection increases it significantly. ***—p<0.001, *—p<0.05.

The presence of *Turneriella* OTU4017244 in the naturally arising tumorous polyps caused multiple morphological alterations and developmental disturbances. While in control polyps the ectodermal muscular fibers are highly organized and strictly oriented along the oral-aboral body axis [23] (Fig 3A), in tumorous polyps these actin filaments are greatly disorganized and lay even perpendicular to the main body axis (Fig 3C). Furthermore, consistent with our previous observations [1], numerous female-committed germline precursor cells positive for the periculin marker [24] were present in the tumorous *H*. *oligactis* polyps, but absent from control polyps (Fig 3B and 3D). Additionally, in tumor-bearing polyps the thickness of the mesoglea between the ectoderm and endoderm had doubled (S5A Fig) and the total number of epithelial cells per polyp had increased two-fold compared to controls (S5B Fig). Tumor-bearing polyps also had more tentacles (on average 12 per polyp) compared to the control polyps (6 per polyp, Fig 3I and 3J). Consistent with our previous findings [1], asexual growth by budding was retarded in tumor bearing polyps (Fig 3K), most likely due to an increased time of bud detachment (Fig 3L). Strikingly, all morphological and developmental changes associated with the presence of spirochetes were reversed by the antibiotic treatment of tumorous polyps (Fig 2C): actin filaments became properly organized (Fig 3E), periculin-positive germline cells almost disappeared (Fig 3F), number of tentacles (Fig 3J) and growth dynamics (Fig 3K and 3L) returned to values characteristic for control polyps.

To better understand the impact of spirochetes on these morphological changes, we next injected a pure culture of *T*. *parva* into healthy control polyps (Fig 3G, 3H and 3J–3L). Remarkably, the addition of this single bacterium was sufficient to trigger all the tumor-specific phenotypic alterations (Fig 2A): actin filaments became disorganized (Fig 3G), germline precursor cells accumulated (Fig 3H), tentacle number increased (Fig 3J), asexual reproduction declined even more than in the naturally tumorous polyps (Fig 3K), and the bud detachment time increased compared to the healthy control (Fig 3K and 3L). Since budding rate and bud detachment time are essential parameters for asexual reproduction, the fitness costs for *H*. *oligactis* to carry spirochetes are substantial. The complete restitution of the tumorous phenotype by solely the introduction of *T*. *parva* into healthy polyps provides an additional evidence for the essential role of spirochetes in tumor formation.

We also note a consistent increase in the interindividual variation associated with tumor-formation. The clonal culture of healthy *H*. *oligactis* demonstrates a very low interindividual variation, evident in minimal deviations in the number of tentacles (Fig 3J), bud detachment time (Fig 3L), mesoglea thickness (S5A Fig) and number of epithelial cells (S5B Fig). This is not surprising, since the number of tentacles and pattern of their emergence as well as the number of cells per polyp are species-specific traits and are genetically controlled [25–27]. In tumorous polyps, interindividual variation is markedly higher compared to the healthy polyps. It is evident in a broad range of tentacle number (Fig 3J), bud detachment time (Fig 3L), mesoglea thickness (S5A Fig) and epithelial cell number (S5B Fig) variance. This variation clearly indicates a loss of a developmental control in tumorous polyps and suggests that tumorigenesis greatly affects morphogenesis. This interindividual variation among tumorous polyps is most likely due to a gradual development of the tumorous phenotype. Supporting this view, the progressive acquisition of the tumorous phenotype with conspicuous tissue bulges and increased tentacle numbers takes several weeks (S2 Fig, S6A Fig). These gradual phenotype changes correlate with a progressive increase in density of spirochetes in the tissue (S6B Fig). Remarkably, the microbiota composition in tumorous polyps is also more variable compared to the healthy polyps (S7 Fig). Particularly, the relative abundance of Gammaproteobacteria, to which the *Pseudomonas* colonizer belongs, substantially differs between polyps. Taken together, our observations uncover a high interindividual variation associated with the tumor phenotype and strongly suggest that the dynamic interactions within the microbiota are causing this variability.

### A commensal bacterium promotes tumorigenesis

**Fig 4:**
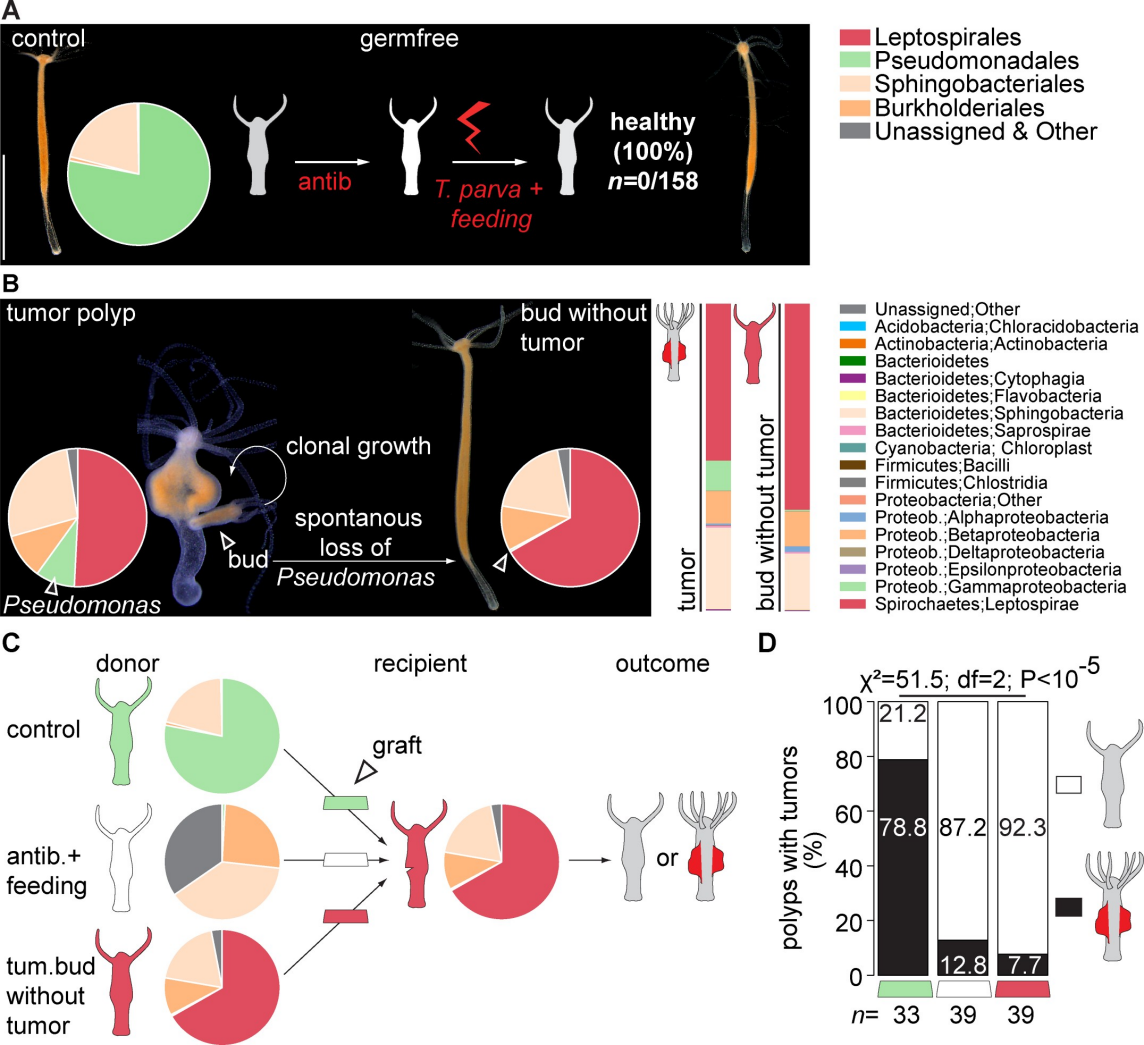
Spirochetes are not sufficient to cause tumorigenesis. **(A)** Presence of some bacteria on *Hydra* is necessary for *T*. *parva* to colonize the host. *T*. *parva* injected into antibiotic-treated polyps free of bacteria were not able to settle, and neither of injected polyps (0/158) developed tumorous phenotype. **(B)** In rare cases, tumor-bearing polyps generate buds that result in healthy tumor-free polyps. These polyps harbor a microbiota dominated by spirochetes, but devoid of Gammaproteobacteria, indicating the loss of *Pseudomonas*. Insets: Abundance plots of the microbial composition of parental tumorous polyps (tumor) and spontaneously recovered polyps (bud without tumor) on the bacteria class level. **(C)** Setup of the transplantation experiment to prove that both bacteria, *T*. *parva* and *Pseudomonas*, are necessary and sufficient to elicit tumorous phenotype in *H*. *oligactis*. A tissue fragment from a healthy donor polyp (control) provides a source of *Pseudomonas*. This graft, or the grafts from an antibiotic-treated polyp devoid of *Pseudomonas* and spirochetes (antib.+feeding), or a tissue fragment from a spontaneously recovered polyp enriched in spirochetes but free from *Pseudomonas* (tum. bud without tumor) are transplanted onto tumor-free recipients devoid of *Pseudomonas* but harboring spirochetes. The tumor formation in resulting polyps is evaluated. **(D)** While only a small fraction of the recipients grafted with only *T*. *parva* (red), or no bacteria (white) on the ECM develop tumors, the recipients with grafts from tissue with *Pseudomonas* (green) have the highest rate of tumor formation.

The above studied effects indicate that the presence of *Turneriella* spirochetes is necessary for tumor growth (Figs 2 and 3). To test whether this spirochete bacterium by itself is sufficient in the absence of other microbes to elicit tumorigenesis, we performed two experiments. First, we injected a pure culture of *T*. *parva* into germ-free control polyps. Unexpectedly, none of the 158 injected polyps developed tumors (Fig 4A). PCR amplification of *T*. *parva* 16S rDNA confirmed that the spirochetes were absent in these polyps (S8 Fig) suggesting that in the absence of other bacteria the spirochetes cannot colonize the hydra tissue. Noteworthy, while *Pseudomonas* was totally absent in these polyps, some other microbes including Burkholderiales and Sphingobacteriales bacteria (Fig 2C) were present in the injected polyps; they most likely were introduced by feeding the polyps with non-sterile *Artemia* nauplii. The specific absence of *Pseudomonas* led us speculate that the presence of this bacterium in the mesoglea of *Hydra* is necessary for *T*. *parva* to settle and subsequently to promote tumor development. To test this hypothesis directly, we first made attempts to colonize the germ-free *Hydra* polyps with *Pseudomonas* and subsequently to introduce the spirochetes. However, all efforts to achieve a stable mono-association with *Pseudomonas* failed for not yet clear reasons.

In a second experiment, we used the tumor-free polyps that spontaneously appeared in the culture of tumorous *H*. *oligactis* polyps as a result of budding (Fig 4B). While most of the buds produced by tumor-bearing polyps develop tumors [1], very rarely some buds emerge and detach from tumor-bearing polyps that remain healthy and do not develop tumorous phenotypes over months (Fig 4B). Analysis of the microbiota composition of these tumor-free buds revealed, unexpectedly, that *Pseudomonas* OTU750018 was virtually absent, with an abundance below 0.5% (Fig 4B), suggesting a spontaneous loss of this bacterium in the developing buds. This is consistent with a high intraindividual variation in *Pseudomonas* abundance (S7 Fig). Since *Turneriella* OTU4017244 was even more abundant in these tumor-free polyps compared to the parental tumor-bearing polyps (Fig 4B), the presence of the *Turneriella* spirochete alone is not sufficient to elicit tumorigenesis, and a substantial amount of *Pseudomonas* bacteria may be needed for tumor formation. Because the relative abundance of *Turneriella* OTU4017244 increased significantly in these *Pseudomonas*-depleted buds compared to the parental culture (Fig 4B), the spirochete seems to occupy niches normally populated by *Pseudomonas*.

To further elucidate the interplay between *Pseudomonas* and *Turneriella*, next we performed transplantation experiments (Fig 4C and 4D). Transplanting tissue with natural microbiota dominated by *Pseudomonas* and devoid of spirochetes from control polyps into tumor-free polyps densely populated by spirochetes and lacking *Pseudomonas* (Fig 4C) caused in most cases (78.8%) tumor formation (Fig 4D). PCR amplification of *Tureneriella* and *Pseudomonas* 16S rDNA confirmed the presence of the both bacteria in the newly formed tumor-bearing polyps (S9 Fig). Polyps that did not develop tumors were always lacking *Pseudomonas* bacteria. Grafting tissue fragments of polyps lacking *Pseudomonas* into tumor-free polyps densely populated by spirochetes only rarely resulted in tumor outgrowth (Fig 4C and 4D). This was demonstrated, first, by transplanting tissue from polyps with a greatly altered microbiota due to antibiotics treatment followed by non-sterile feeding (Fig 4A) into tumor-free polyps densely populated by *Turneriella* spirochetes (Fig 4C), and second, by transplanting *Pseudomonas*-free but spirochete enriched tissue from buds into tumor-free polyps densely populated by spirochetes (Fig 4C). Both transplantation approaches resulted in a very low level of tumor formation compared to transplanting *Pseudomonas*-containing tissue (7.7% and 12.8% compared to 78.8%, Fig 4D). This suggests that the common resident bacterium *Pseudomonas* plays a crucial and synergistic role in tumorigenesis and somehow interacts with the spirochete *Turneriella*.

### Tumor induction by a phylogenetically distant spirochete, *Leptospira* sp. from *H*. *circumcincta*

Our experiments indicated that the presence of spirochete bacteria is necessary for tumor formation, yet not sufficient. Only if a strain of *Pseudomonas* colonizes the same niche–the mesoglea of *Hydra*, the tumor formation is triggered. To provide an additional evidence that the presence of both bacteria in the ECM of *Hydra* is sufficient for tumorigenesis, we made use of another *Hydra* species—*H*. *circumcincta*. According to our previous studies [15], polyps of this species have spirochetes in their microbiota (S10A Fig). Microscopic analysis confirmed the localization of numerous spirochete cells in the mesoglea (S10B Fig). The phylogenetic analysis of 16S rRNA gene sequence identified this spirochete as a member of Leptospiraceae family, however phylogenetically distant from *Turneriella* OTU4017244 of *H*. *oligactis* and *T*. *parva* DSM21527 (S10C Fig). Notably, no *Pseudomonas* bacteria could be detected in the mesoglea of *H*. *circumcincta* host, and tumors have never been reported in this *Hydra* species. In order to test the tumorigenic potential of the spirochete from *H*. *circumcincta*, we introduced this bacterium into the mesoglea of healthy *H*. *oligactis* polyps that normally harbor *Pseudomonas* in their mesoglea (S10D Fig). In 20% of the cases, this “xeno”-transplantation resulted not only in a successful colonization of the foreign spirochetes but also in subsequent tumor formation (S10D Fig). Taken together, these observations provide strong evidence that colonization of the extracellular matrix (mesoglea) of *Hydra* by both, *Pseudomonas* strain OTU750018 and spirochete bacteria such as *Turneriella* OTU4071244, *T*. *parva* DSM21527 and *Leptospira* from *H*. *circumcincta*) is sufficient to elicit tumorigenesis. Evidently, in the absence of *Pseudomonas* in the mesoglea, the tumorigenic potential of the spirochetes cannot be expressed.

### Insights from the *Pseudomonas* and *Turneriella* genomes

**Fig 5:**
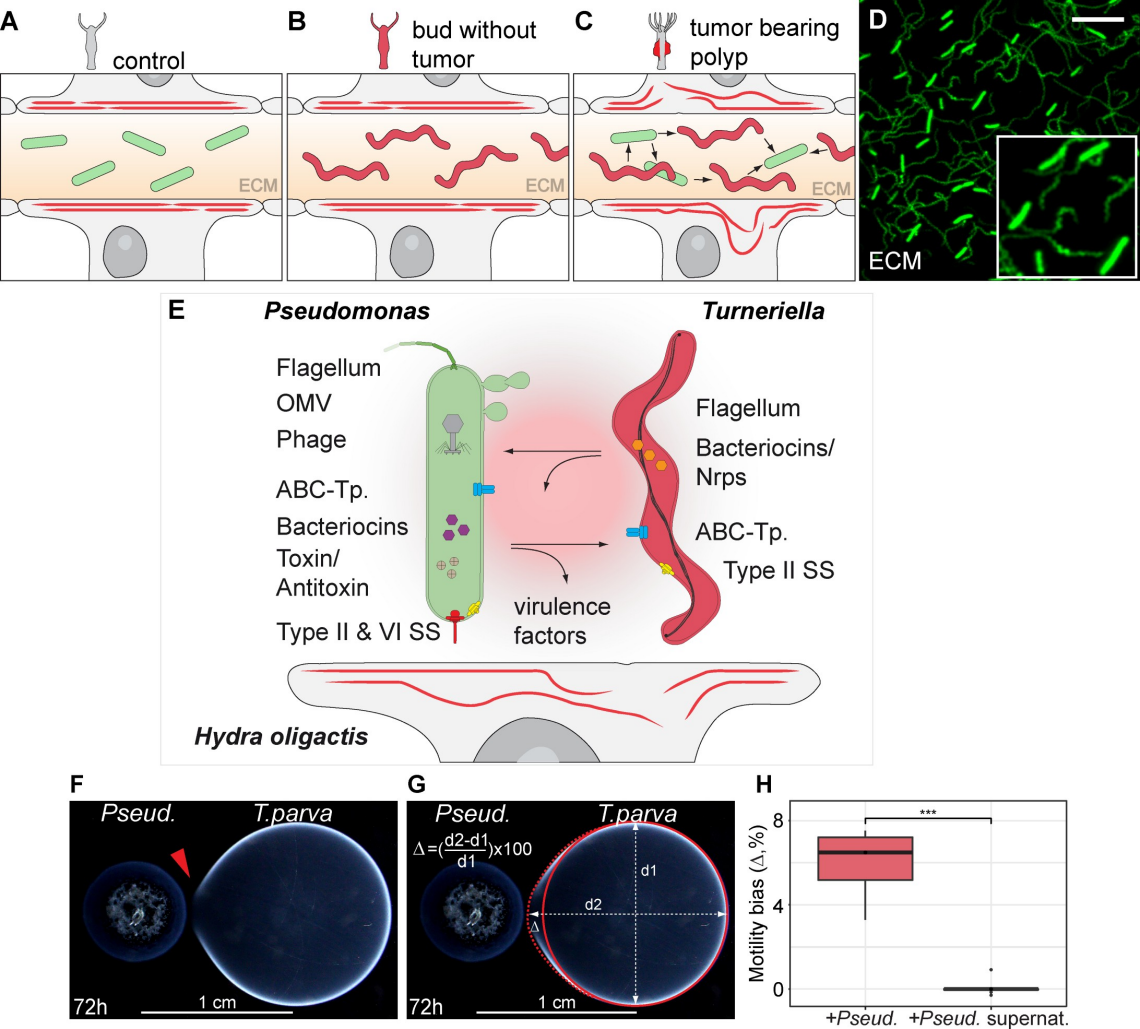
Interplay between the commensal *Pseudomonas* and environmental spirochetes within *Hydra* mesoglea induces tumor formation. **(A)** In healthy *H*. *oligactis* polyps the mesoglea (ECM) is colonized only by *Pseudomonas*, actin cytoskeleton of the epithelial cells (red lines) is well organized and no developmental abnormalities can be observed. **(B)** In the polyps that spontaneously lost *Pseudomonas*, the mesoglea is colonized only by *T*. *parva*, and the phenotype is normal. **(C)** If the both bacteria, *Pseudomonas* and *Turneriella*, are present in the ECM of a polyp, an interaction between them occurs and likely causes actin fiber disorganization (red curved lines) and tumor outgrowth. **(D)** In the tumor-bearing polyps, both bacteria–rod-shaped *Pseudomonas* and helical-coiled *T*. *parva*, are found in close proximity in the ECM (revealed by SYBR-gold staining; scale bar: 5 μm), suggesting an direct physical interaction. **(E)** Analysis of genome sequences from the *Pseudomonas* OTU750018 isolated from *H*. *oligactis* and *T*. *parva* reference strain DSM21527 uncovers their repertoires of putative virulence factors. In *Pseudomonas* genome (left), genes coding for the entire flagellum assembly, type II and VI secretion systems (Type II & VI SS) and the Sec-SRP complex are detected along with multiple ABC-transporters (ABC-Tp), bacteriocins, and toxin/antitoxin systems. Additionally, a complete prophage (Phage) is integrated into the *Pseudomonas* genome. Electron microscopy analysis (S15 Fig) also suggests that *Pseudomonas* cells release outer membrane vesicles (OMVs). The genome of *T*. *parva* (right) harbors genes coding for flagellum assembly machinery, few bacteriocin-like proteins and several ABC-transporters (ABC-Tp), few genes coding for components of type II secretion system (Type II SS) and Sec-SRP complex. We speculate that the interplay between two bacteria (arrows) and some virulence factors produced by them affect the *H*. *oligactis* cells, their morphology (including the actin cytoskeleton depicted as red curved lines), disturb the tissue homeostasis and cause tumor formation. **(F)** Evidence for direct interaction between *T*. *parva* and *Pseudomonas*. In the presence of *Pseudomonas* cells, motility of *T*. *parva* increases, as evidenced by a prominent colony protrusion toward the Pseudomonas (red arrow). **(G)** The motility bias parameter Δ was used to quantify the asymmetric motility of *T*. *parva*. (H) *T*. *parva* demonstrates a positive motility bias only in the presence of living *Pseusomonas* cells (+Pseud.). Cell-free supernatant of *Pseudomonas* culture (+Pseud. supernat.) does not cause motility bias in *T*. *parva*. ***—p<0.00.1. See also S17 Fig.

Our results suggest that an interaction between the environmental spirochete bacterium *Turneriella* and the commensal *Pseudomonas* drives the tumorigenesis in *H*. *oligactis* (Fig 5A–5E). In order to gain insights into the molecular mechanisms of the interactions between the two bacteria, we first sequenced the genome of the resident bacterium *Pseudomonas*. For this, *Pseudomonas* OTU750018 was isolated from the mesoglea of *H*. *oligactis* control polyps, cultured *in vitro*, and its genomic DNA was isolated and sequenced using the MiSeq platform (Illumina). We annotated the gene content in the *Pseudomonas* genome using multiple databases (see Methods, S1 Data, S2 Table). Analysis of the *Pseudomonas* genome revealed the presence of, among others, genes coding for the entire flagellum assembly (Fig 5E, S11A Fig), type II and VI secretion systems, and the Sec-SRP complex (S12 Fig) as well as multiple ATP-binding cassette (ABC) transporters (S13 Fig), bacteriocins, and toxin/antitoxin systems (S3 Table). These factors are of particular interest since they may be involved in the interaction of *Pseudomonas* with both the spirochetes and the *Hydra* host. The flagellum and pili [28–34], diverse secretion systems [35–37] as well as ABC-transporters [38–40] have been previously reported as virulence factors crucial for colonization, persistence and pathogenesis of diverse Gram-negative bacteria, including the closely related *P*. *aeruginosa* species. These factors promote bacterial virulence not only by enhancing attachment to eukaryotic cells, but also by mediating the delivery of secreted effector proteins (toxins) from the cytosol of the bacteria into host cells. They may also activate the innate immune response of the host and mediate interactions between bacteria. The discovery of a complete prophage integrated into the *Pseudomonas* genome (Fig 5E, S14 Fig, S4 Table, S1 Data) points to additional tools to interact with the spirochetes. Furthermore, several genes known to be essential for the formation of outer membrane vesicles (OMVs) on the bacterial surface were found in the *Pseudomonas* genome (Fig 5E). Consistently with that, high-resolution electron microscopy analysis uncovered conspicuous OMVs on the surface of *Pseudomonas* bacteria colonizing the extracellular matrix (S15 Fig). Substances released by means of OMVs are known to digest the bacteria environment or even affect the eukaryotic host cells by releasing toxins [41–43] Finally, multiple genes encoding secreted enzymes, such as collagenases and hydrolases, were detected (S2 Table). These findings are consistent with the observation (S1 Fig) that *Pseudomonas* cells are always located in the mesoglea within lacune-like electron transparent areas free from fibrillary components. Hence, *Pseudomonas* appears to actively digest the extracellular matrix of *Hydra*. Taken together, these findings demonstrate that *Pseudomonas* possesses a rich repertoire of factors that may play a critical role in the interaction with both, the spirochetes and the *Hydra* host (Fig 5E).

To perform a similar study in *Turneriella* spirochetes, we analyzed the genome of *T*. *parva* DSM21527 that has been previously sequenced [22]. Importantly, this strain is phylogenetically very close to the native *Turneriella* strain found in *H*. *oligactis* tumors (Fig 1H) and is also capable of eliciting the tumorous phenotype in the same manner as the native strain (Figs 2 and 3 and S2 Fig). In contrast to *Pseudomonas*, the *T*. *parva* genome contains relatively few putative virulence factors (Fig 5E). Our analysis uncovered only the genes coding for flagellum assembly (S11 Fig, S5 Table, S2 Data), few bacteriocin-like proteins (S6 Table) and several ABC-transporters (S13 Fig) as well as putative collagenase and hydrolase enzymes. The genes coding for components of the type II secretion system and Sec-SRP complex were only partially represented in the spirochete genome (S12 Fig).

In sum, *Pseudomonas* and *T*. *parva* both possess a variety of putative virulence factors that could mediate the interactions between them and the *Hydra* host resulting in impaired tissue homeostasis and tumorigenesis (Fig 5E).

### Evidence for interaction between *Pseudomonas* and *Turneriella*

In order to provide a direct evidence that *Pseudomonas* and *Turneriella* do interact, we performed several experiments. First, we estimated the motility of both, *Pseudomonas* OTU750018 isolate and *T*. *parva* DSM21527 strain in a swarming assay (Fig 5F–5H, S16 and S17 Figs). Both bacteria demonstrated a prominent motility on semi-solid agar and, if plated alone, spread as perfectly round colonies with sharp borders (S16A and S16B Fig). Remarkably, if both bacteria were inoculated on the same spot into the agar, the motility of both was significantly increased (S16C and S16D Fig) with the diameter of the *T*. *parva* colony being over 60% larger in the presence of *Pseudomonas* compared to a *T*. *parva* colony grown alone (S16C and S16D Fig).

Second, when *Pseudomonas* OTU750018 and *T*. *parva* DSM21527 were spotted onto the same plate at a distance 10 mm between each other the spreading of *T*. *parva* colony became strongly biased towards the *Pseudomonas* culture (Fig 5F, S17A–S17C Fig). The conspicuous growth protrusion directed towards the *Pseudomonas* colony may indicate a substantially increased motility of the spirochete cells induced by *Pseudomonas*. Importantly, this phenomenon was not observed in the presence of a sterile-filtered supernatant from *Pseudomonas* culture (Fig 5H, S17D Fig), indicating that only living *Pseudomonas* cells can alter the spirochetes’ motility. In the presence of another *T*. *parva* culture spotted onto the same culture dish, the motility of *T*. *parva* was not affected either (S17E Fig) and both colonies spread as completely circular until they merged in less than 72 hours (S17E and S17F Fig). This clearly indicates that an interaction between two bacteria accompanied by increased motility takes place *in vitro*.

Last, we monitored the motility of individual *Pseudomonas* OTU750018 and *T*. *parva* DSM21527 cells using phase-contrast microscopy (S18 and S19 Figs, S1–S6 Movies). *T*. *parva* demonstrates a relatively slow and undirected swimming motility (S18A Fig, S1 Movie) similar to that described in closely related *Leptospira biflexa* species [44]. In contrast, *Pseudomonas* cells display a rather fast directed swimming behavior (S18B Fig, S2 Movie), likely assisted by its flagellum and clearly different from the previously described twitching motility characteristic for *P*. *aeruginosa* [45]. Interestingly, in the presence of a *Pseudomonas* cell, the motility of *T*. *parva* changes to a directional migration towards *Pseudomonas* cells (S19A and S19B Fig, S3 and S4 Movies) resulting in a physical contact between the both bacteria. These interactions were either transient (S19A Fig, S3 Movie), recurrent (S19B Fig, S4 Movie), or permanent, whereby the two bacteria became firmly attached to each other and floated jointly in the same direction (S19C and S19D Fig, S5 and S6 Movies). Taken together, these data provide direct evidence that two bacteria do interact and also alter their behavior upon physical contact.

**Fig 6:**
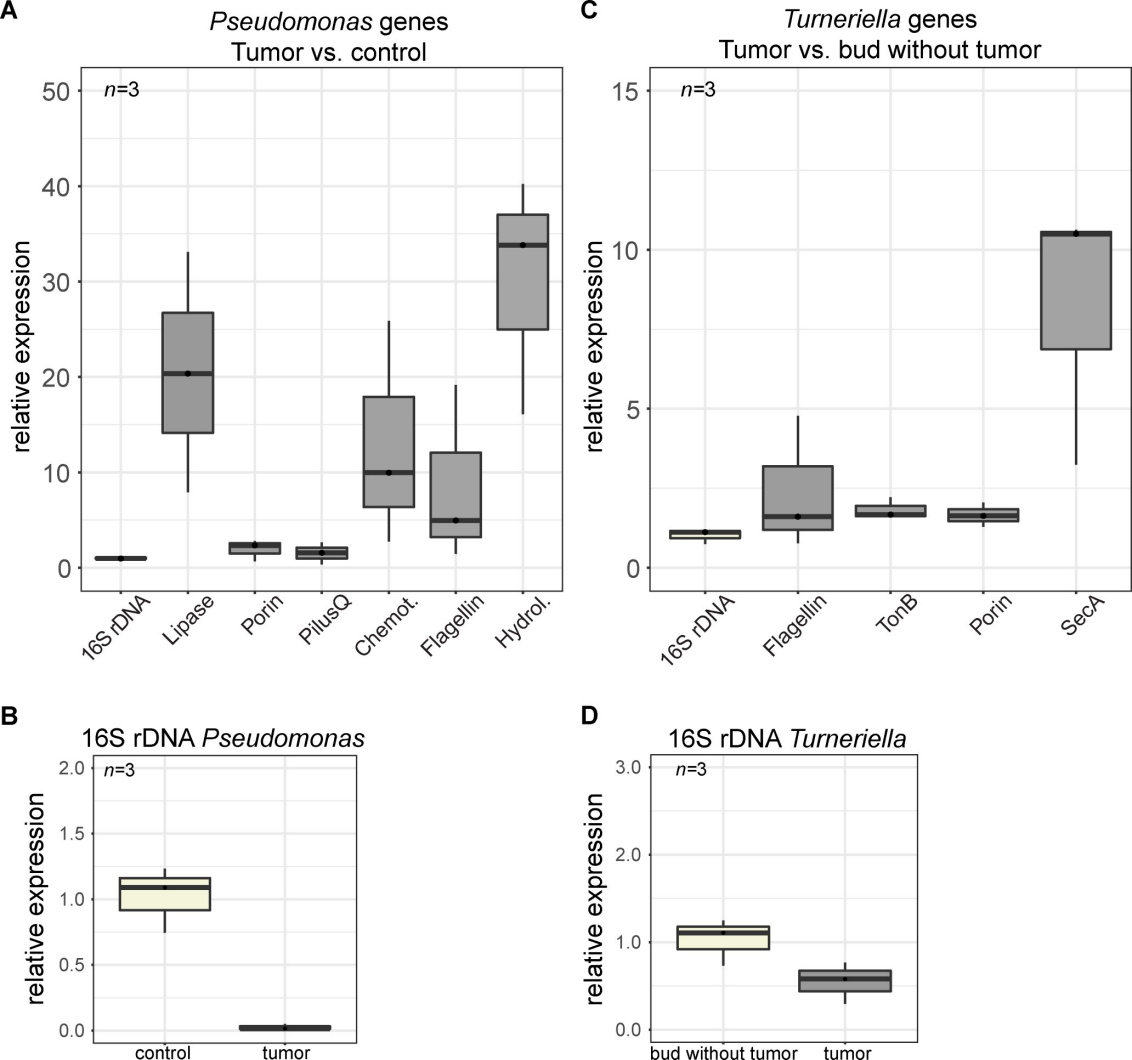
Multiple putative virulence factors *Pseudomonas* and *Turneriella* are up-regulated in tumor context. **(A)** Expression of six genes encoding putative virulence factors of *Pseudomonas* in tumor polyps compared to healthy control polyps. The relative expression values are normalized to *Pseudomonas* 16S rDNA expression level to account for a differential abundance of *Pseudomonas* in tumorous and control polyps. Consistently with RNA-seq analysis (S6 Table), these six virulence factors are enriched in tumorous tissue, i.e. their expression in *Pseudomonas* is activated by the presence of *Turneriella*. **(B)** The density of *Pseudomonas* in tumorous polyps is dramatically lower than in healthy control polyps, consistent with earlier observations (Fig 1C, S6 Fig). **(C)** Expression of four genes coding for putative virulence factors of *Turneriella* in tumorous polyps compared to polyps that spontaneously lost tumors (Fig 4B). The relative expression values are normalized to *Turneriella* 16S rDNA expression level to account for a differential abundance of spirochetes in tumorous and polyps free of tumors with dramatically reduced *Pseudomonas* density (Fig 4B). Consistently with RNA-seq analysis (S6 Table), the virulence factors are enriched in tumorous tissue, i.e. their transcription in *Turneriella* is activated in the presence of *Pseudomonas*. **(D)** The density of *Turneriella* in tumorous polyps is slightly lower than in healthy polyps that spontaneously lost Pseudomonas and tumors, consistent with earlier observations (Fig 4B).

In order to gain insights into the molecular mechanisms behind these interactions that ultimately may lead to tumor formation, we analyzed the dynamics of gene expression in the both bacteria in the tumor context. First, we performed a metatranscriptome analysis of the tumorous *Hydra* tissue colonized by both, *Turneriella* OTU4017244 and *Pseudomonas* OTU750018, and also of healthy control tissue that harbors only *Pseudomonas*. Among the top 50 genes differentially expressed by both bacteria in the tumor context, we discovered transcripts for multiple genes coding for flagellar proteins, secretion system assembly machinery, porin-like toxins and enzymes (S7 Table). Interestingly, some other genes present in the genomes of both *Pseudomonas* and *Turneriella* (S2–S6 Tables), were not present in the transcriptome and thus are unlikely involved in tumorigenesis. To validate the differential expression of these candidate genes in the tumor context, we performed qRT-PCR experiments (Fig 6). Consistently with the RNA-seq analysis, multiple genes coding for putative virulence factors were up-regulated in the tumorous tissue (Fig 6) including a flagellin gene which was transcribed in *Pseudomonas* in the presence of *Turneriella* (Fig 6A). These expression data strongly support our *in vivo* motility observations (Fig 5F, S16C and S16D Fig, S17 Fig). Additionally, *Pseudomonas* genes coding for a hydrolase, lipase, a chemotaxis protein as well as a porin were strongly up-regulated in the tumorous polyps (*i*.*e*. in the presence of spirochetes) compared to controls (*i*.*e*. without spirochetes; Fig 6A). This indicates that these virulence genes are specifically activated in the tumor context and in the presence of spirochetes. Similarly, several transcripts coding for putative virulence factors of *Turneriella*, such as flagellin, porin, TonB and SecA protein, were specifically enriched in the tumor context, (Fig 6C). Taken together, these observations provide strong support for the view that interaction between *Pseudomonas* and spirochetes causes a change in behavior and also in gene expression of putative virulence factors.

### Stress facilitates spirochete infection

**Fig 7:**
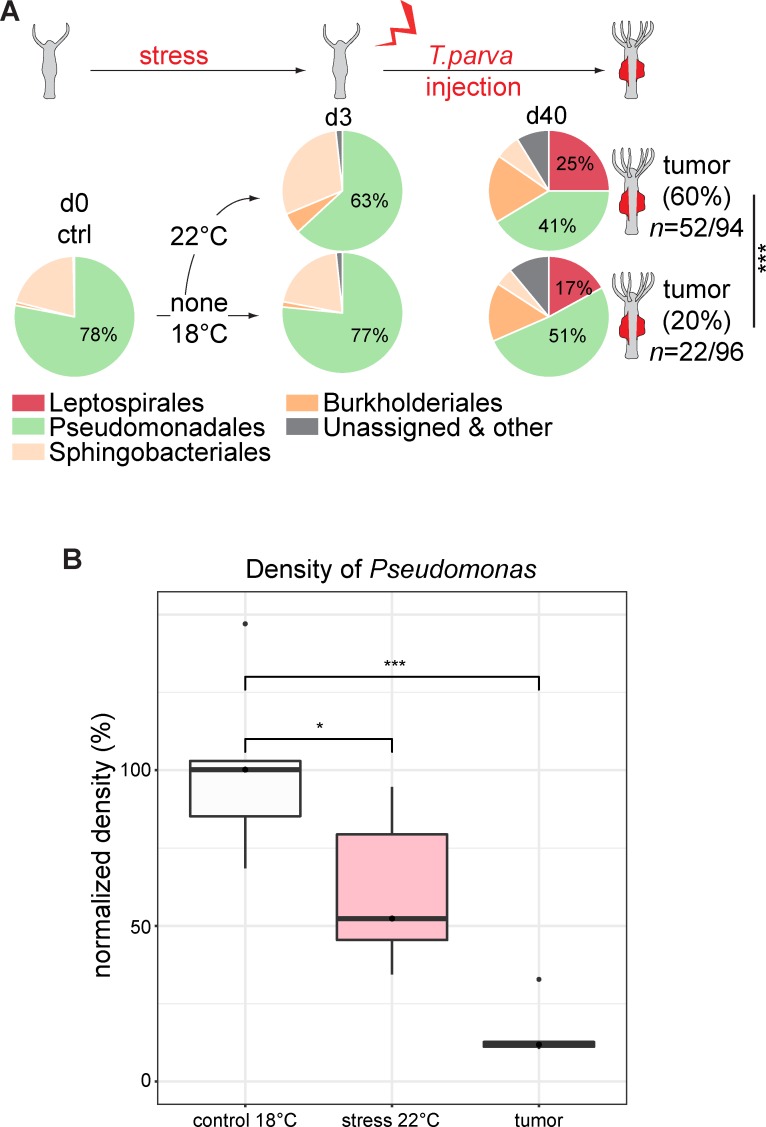
Temperature stress facilitates colonization of *H*. *oligactis* by *T*. *parva* and tumor formation. **(A)** *T*. *parva* injected into *H*. *oligactis* colonizes the polyps more successfully, reaches higher abundance and induces tumors more frequently if the host was pre-stressed with elevated temperature (22°C). The microbiome of healthy control *H*. *oligactis* changes substantially after 3-day treatment of polyps at 22°C. Relative abundance of bacteria identified by 16S rDNA sequencing and resolved at the phylum level, *n = 6*. A successful injection rate with tumorous outcome is higher (52 of 94 injected polyps) in pre-stressed animals (22°C) compared to untreated animals (22 of 96 injected polyps) (chi-square statistics: χ^2^ = 20.9; df = 2; P<10–5). **(B)** Temperature stress results not only in a decrease of *Pseudomonas* OTU750018 relative abundance, but also in a significant decrease of density (absolute abundance). In the tumorous polyps, the abundance of Pseudomonas is even lower. The density of *Pseudomonas* colonization was estimated using qRT-PCR amplification of *Pseudomonas* OTU750018 16S rDNA gene and normalized to the values in the healthy control polyps at 18°C. gDNA was extracted on day 3 of temperature treatment, before injection of *T*. *parva*.

Because tumor formation in *H*. *oligactis* is a rather rare event [1] and *Turneriella* bacteria are normally not found in association with *H*. *oligactis* species (Fig 1A) [15] but are reported to be present in tap water [46,47], the normal *Hydra* epithelium with its resident microbiome appears to efficiently protect against spirochete–induced tumorigenesis. To determine if this natural protection gets lost under environmental stress, we challenged control *Hydra* polyps with high temperature (22°C for 3 days, Fig 7A). Interestingly, this had a strong impact on the microbiota and resulted in a significantly reduced relative abundance as well as absolute abundance (density) of *Pseudomonas* in temperature-stressed polyps (Fig 7A and 7B). Subsequent injection of *T*. *parva* into the temperature-stressed polyps resulted in much higher colonization rates and tumor outcomes (60%, Fig 7A) compared to injection into intact polyps (20%, Fig 7A) indicating that a reduced number of *Pseudomonas* cells may open up a niche for the spirochetes to settle. These data also could provide a cue for how the tumor might have originated in the mass culture of *H*. *oligactis* over 10 years ago [1]. We assume that an undefined environmental stress may have reduced the density of *Pseudomonas* in an otherwise healthy and well protected *H*. *oligactis* polyps and allowed the ubiquitously present spirochetes to colonize the founder polyp.

## Discussion

This study emphasizes how little we know about the role of the resident microbiome in maintaining tissue homeostasis and pathogen defense, a fundamental process that is likely to take place in every tissue of every animal species. Our data have unveiled that *Turneriella* colonization is essential for triggering the complex tumorous phenotype in *Hydra* (Figs 1–3). Surprisingly, *Turneriella* is not sufficient to cause tumorigenesis and the simultaneous presence of resident *Pseudomonas* bacteria in the same niche, the mesoglea (ECM), is essential for the outbreak of the disease (Fig 4C). Although the genomes of both *T*. *parva* and *Pseudomonas* contain a number of virulence factors (Fig 5E), both bacteria appear to be innoxious if they colonize the *H*. *oligactis* mesoglea alone. The pathogenic potential is only realized when both bacteria are present at the same time. Hence, the pathogenicity of *Pseudomonas*, a natural member of the *H*. *oligactis* microbiota, is strictly context-dependent; it is strongly dependent on the presence of the *Turneriella* spirochete. A similar context-dependent activation of virulence and shift from host-commensal to host-pathogen interaction has been reported previously [48–50] for *P*. *aeruginosa* which in the pathogenic state may act on the actin cytoskeleton of the host cells [51,52].

Our observations clearly indicate that *Pseudomonas* and the spirochetes elicit tumorigenesis synergistically, suggesting a molecular interplay to take place between these two colonizers. Our *in vitro* motility assays and gene expression analysis provide direct evidence for this interaction to take place. Although the precise mechanism of this interaction remains to be uncovered, secreted molecules (as suggested by the agar motility assay, S17 Fig) and a physical interaction (S19 Fig, S3–S6 Movies) are likely to be involved. Since expression of hydrolases and collagenases is induced when both bacteria are present in the mesoglea (Fig 6, S7 Table), and a local degradation of the extracellular matrix may affect its stiffness and thus the cytoskeletal organization of eukaryotic cells [53–55], one plausible scenario could be that active degradation of the ECM by *Pseudomonas* and/or *Turneriella* (S1 Fig) elicits actin fiber disorganization in *Hydra’s* epithelial cells ultimately resulting in loss of tissue homeostasis and multiple developmental defects (Fig 5E). It also is conceivable that some virulence factors or toxins are released by OMVs (S15 Fig) or directly delivered into the host cells through a secretion system [56]. In sum, here we show that in the holobiont *Hydra* [57] interactions between two bacteria, *Pseudomonas* and *Turneriella*, have profound effects on tissue homeostasis and fitness. Considering that *Turneriella* is an environmental bacterium present in pond water, these findings highlight the role of environment as a source (reservoir) of opportunistic pathogenic microbes, that, upon favorable conditions, may colonize a novel habitat, *i*.*e*. the multicellular host, and cause adverse effects on its development, physiology and fitness.

A causal connection between microbiota and cancerogenesis is being increasingly appreciated [58,59]. Several tumors in humans are strongly associated with pathogenicity of a single bacterium, such as *Helicobacter pylori* [60], or are likely caused by interactions between two commensal bacteria, like *Bacteroides fragilis* and *Escherichia coli* [12]. Additionally, there is an accumulating evidence that commensal bacteria may support tissue homeostasis by, for instance, stimulating neurogenesis [61,62]. These observations point to the evolutionary conserved role of the host-associated bacteria in maintaining host tissue homeostasis. The basal phylogenetic position of *Hydra* allows considering the tumorigenic potential of bacteria in an evolutionary context. Better understanding of the protective function of the resident microbiome in *Hydra* may thus help refine current concepts of tumorigenesis, with important implications for early diagnosis and therapy. Last, our findings may also stimulate thoughts about the link between microbiota, antibiotics and cancer. Antibiotics not only have substantial direct and indirect effects onto microbiota [63,64], they may also represent a novel strategy for prophylaxis and/or treatment of certain cancer types.

## Materials and methods

### Animals and culture conditions

Experiments were carried out using *H*. *oligactis* strain St. Petersburg. Animals were maintained under constant environmental conditions, including culture medium (HM), temperature (18°C) and food according to standard procedures [65] (twice a week). The propagation of the polyps occurs by only asexual reproduction resulting in clonal cultures. Natural occurrence of tumor polyps has been already described [1]. Tumor buds have been separated long time ago from the original cultures and been kept in tumor only cultures. The fitness loss in tumor polyps is still recognizable but constant feeding results in slow and ongoing asexual reproduction. The majority of the buds attain the tumor phenotype after around 4 weeks. In rare cases, buds do not exhibit the tumor phenotype and remain healthy. These specific buds were separated and kept apart from the parental cultures. In this study, work has only been performed with the *H*. *oligactis* species. *Pelmatohydra robusta* tumor cultures are intact but have not been of interest in this work.

### Bacteria culture conditions and generation of germ-free *Hydra*

To obtain germ-free (gf) *H*. *oligactis*, polyps were kept for 2 weeks in an antibiotic solution containing 50 μg/ml each of ampicillin, rifampicin, streptomycin, spectinomycin and neomycin with exchange of the solution every second day. Post treatment polyps were transferred into sterile-filtered HM for one week which was changed every second day. The absence of bacteria was verified by a 30-cycle PCR using the universal 16S bacteria Primer EUB_27F and EUB_1492R [66], whereas the positive control of none-treated (DMSO) polyps showed a signal. Because *T*. *parva* was uncultivable, the closest relative *T*. *parva* [46] DSM 21527 was ordered and kept in Leptospira semisolid medium which was produced and handled based on the manufactures protocol (Difco Leptospira Medium Base EMJH & Difco Leptospira Enrichment EMJH; Thermo Fisher Scientific, USA). *T*. *parva* was inoculated twice a week to new media to keep the culture alive.

### Isolation of the mesoglea

Isolation of the *Hydra* mesoglea was performed as previously described [67]. Adapted from the protocol IGEPAL CA-630 was used (1:1000 in ddH_2_O, Sigma-Aldrich, Germany) to separate the mesoglea from the *Hydra* cells. Isolated mesoglea was used for the isolation of *Pseudomonas* and SYBR-gold stainings.

### Isolation of *T*. *parva*

We attempted to isolate a pure culture of *Turneriella* OTU4017244 from tumorous *H*. *oligactis* polyps. Although we tested a number of conditions, including the temperature, oxygen concentration, medium composition and addition of selective antibiotics, we never succeeded in obtaining a pure culture. Therefore, we used a closely related culturable strain *T*. *parva* DSM21527 [68] for functional analyses. The bacteria were cultivated in the semisolid Leptospira Base and Enrichment Medium (Difco, purchased from Thermo Fisher Scientific, USA) according to the manufacturer’s instruction. Optimal growth was achieved at 29ºC. To maintain an active *T*. *parva* culture, the bacteria were re-inoculated into a fresh medium every week.

### Isolation of *Pseudomonas*

*Pseudomonas* OTU750018 was isolated from the ECM of *Hydra oligactis* control polyps. First, *H*. *oligactis* polyps were treated with IGEPAL CA-630 [67] to eliminate all bacteria present on the outer surface of the polyps, referred to as glycocalyx, and thus to facilitate the isolation of a clean *Pseudomonas* strain. Further, the mesoglea was inoculated onto R2A-Agar (Carl Roth; Karlsruhe) plates. Finally, *Pseudomonas* OTU750018 bacteria were cloned by repeated plating of single colonies onto R2A-Agar and identified by 16S rDNA Sanger sequencing (IKMB, Kiel).

### Injection of *T*. *parva* in *H*. *oligactis*

Polyps of *H*. *oligactis* were injected with a pure culture of *T*. *parva* DSM21527. The concentration of *T*. *parva* cells was adjusted to 20,000 bacteria per 1μl using a C-Chip Disposable Hemocytometer DHC-N01 (NanoEnTek, USA) and methylene blue cell staining. We injected 5μl of *T*. *parva* suspension containing 100,000 cells into hydra tissue with a fine needle pulled from a glass capillary and connected to a rubber tube with a sterile 0.2 μm filter (Sarstedt, Germany). Polyps were kept in the bacterial medium for 24h and subsequently moved into new dishes with fresh HM. Phenotypes were observed after 4–6 weeks post injection.

### DNA extraction and sequencing of 16S rRNA gene

For total DNA extraction, single polyps (six replicates for each treatment) were washed three times in sterile Hydra-medium and subjected to the DNeasy Blood & Tissue Kit (Qiagen, Germany). Extraction was performed following the manufacturer’s protocol, except that DNA was eluted in 30 μl and kept at -20°C until sequencing. Variable regions 1 and 2 (V1V2) of the bacterial 16S rRNA genes were amplified using the primers 27F and 338R [69]. Bacterial 16S rRNA profiling was performed in the Illumina MiSeq platform with paired-end sequencing of 2x 300bp. The 16S RNA sequencing raw data are deposited at the SRA and are available under the project PRJNA602941. Sequence analysis was executed by using QIIME 1.9.0 package (RRID:SCR_008249) [70]. SeqPrep was used to assemble Paired-end reads (RRID:SCR_013004), chimeric sequences were screened with ChimeraSlayer (RRID:SCR_013283) [69] and manually verified prior to removal from the data set. Sequences were kept in the analysis if they have been present at least in two independent samples.

### Bacterial community analysis

Operational taxonomic units (OTUs) picking was performed using the pick_open_reference_otus.py script with at least 97% identity per OTU. Annotation was conducted using UCLUST algorithm (RRID:SCR_011921) [71] was used to operate the annotation against the GreenGenes database v13.8 (RRID:SCR_002830) [72] implemented in QIIME. To avoid false positive OTUs originated from sequencing errors OTUs with <50 reads were removed from the data set [73]. Read number was normalized to the lowest number of reads in the dataset (8,000). The tables of bacterial abundance were further processed using Linear discriminant analysis effect size (LEfSe) analysis (RRID:SCR_014609) [21] to identify bacterial taxa that account for major differences between microbial communities in tumorous and healthy polyps. To detect bacterial taxa with significant differential abundance, Kruskal–Wallis and Wilcoxon signed-rank sum tests were implemented, and the bacteria were ranked by effect size obtained from linear discriminant analysis (LDA score). P-values were corrected for multiple hypotheses testing by Benjamini and Hochberg’s false-discovery rate correction (q-value). A q-value of 0.001 and an effect size threshold of 4.0 (on a log_10_ scale) were used for all comparisons discussed in this study. The results of LEfSe analysis were visualized by plotting the phylogenetic distribution of the differentially abundant bacterial taxa on the Ribosomal Database Project (RDP) bacterial taxonomy.

### Statistics

Statistical analyses for the 16s rRNA data were performed using two-tailed Student’s t-test or Mann-Whitney U-test if applicable. If multiple testing was performed, p-values were adjusted using the Benjamini-Hochberg correction [74]. Statistics for the fitness measurements were performed using one-way-ANOVA (Kruskal-Wallis test) and an additional Dunnett’s multiple comparison posttest comparing all values to the control.

### Sequencing and annotation of bacterial genomes

For sequencing the *Pseudomonas* OTU750018 genome, a pure culture of this isolate was grown overnight in R2A medium, washed in sterile PBS and subjected to the DNeasy Blood & Tissue Kit (Qiagen, Germany). Nextera XT kit (Illumina) was used for library preparation and paired-end sequencing was conducted on the MiSeq platform (Illumina) at Centre for molecular biology in Kiel. Genome was assembled with Spades 3.12 using default settings. Further, we annotated this genome of *Pseudomonas* OTU750018 as well as the previously sequenced and assembled genome of *T*. *parva* DSM21527 (PRJNA50821) [22]. Several complementary approaches were used to annotate the assembled genome sequences. First annotation was performed with Dfast_core (v.1.0.8). The genes were annotated by aligning the genome sequence with the data previously deposited in diverse protein databases including the National Center for Biotechnology Information (NCBI; https://www.ncbi.nlm.nih.gov/) non-redundant protein (Nr) database, UniProt/Swiss-Prot (https://www.uniprot.org/) and Kyoto Encyclopedia of Genes and Genomes (KEGG; https://www.kegg.jp/ [75]). Additional annotation was carried out using the following databases: Virulence Factors of Pathogenic Bacteria (VFDB; http://www.mgc.ac.cn/VFs/main.htm [76]) and antibiotics and Secondary Metabolite Analysis Shell (anti-smash; https://antismash.secondarymetabolites.org/ [77]). Prophages were identified using the PHAge Search Tool (http://phast.wishartlab.com/, [78]). Nr and GO annotation was carried out using Blast2GO. An E-value of 1e−5 was used as the cut-off for all basic local alignment search tool.

### Metatranscriptome sequencing and analysis

To identify genes of *Pseudomonas* and *Turneriella* transcribed in the *H*. *oligactis* tumor, total RNA was isolated from whole polyps as previously described [1]. After a ribosomal RNA depletion, 15 cDNA libraries (5 for each control, female and tumor animals) were generated and sequenced on the Illumina HiSeq2500 v4 platform, with 125 bp paired-end sequencing of 12 libraries per lane. This resulted in 13–24 million reads per sample after quality control. Quality and adapter trimming was performed using Trimmomatic 0.36 [79]. The RNA-Seq raw data are deposited at the Sequence Read Archive (SRA) and are available under the project ID PRJNA602941. Bowtie2 2.3.5 [80] was used to map the RNA-reads against the gene models of an in-house assembly of the *Pseudomonas* OTU750018 and the publicly published *T*. *parva* DSM21527 genome (PRJNA50821) [22]. The analysis was performed by mapping reads against a combined reference comprising gene models of both *Pseudomonas* and *Turneriella* genomes. Mapping rates ranged around 3% in all samples, resembling the fact that most of the sequenced reads belong to the *Hydra* transcriptome. All downstream analyses were conducted in ‘R’ software [81]. Differentially expressed contigs were identified with the package DESeq2 1.16.1 [82].

### Quantitative real-time PCR analysis (qRT-PCR)

In order to validate the differential expression of putative virulence genes of *Pseudomonas* and *T*. *parva* in the tumor, we performed quantitative real-time PCR analysis. Total RNA was extracted from *H*. *oligactis* polyps and converted into cDNA using First Strand cDNA Synthesis Kit (ThermoFisher Scientific) according to manufacturer’s instruction. Amplification was performed as previously described [83] using GoTaq qPCR Master Mix (Promega, Madison, USA) and specific oligonucleotide primers (S8 Table). Three biological replicates of healthy control *H*. *oligactis*, tumorous polyps and polyps grown from buds without tumor were analyzed with two technical replications. The data were collected using ABI 7300 Real-Time PCR System (Applied Biosystems, Foster City, USA) and analyzed by the conventional ΔΔCt method. To estimate the stress-induced changes in abundance of *Pseudomonas* OTU750018, genomic DNA was isolated from 6 biological replicates of *H*. *oligactis* incubated at 18°C or stressed at 22°C for 3 days, and from tumor *H*. *oligactis* polyps. Real-time amplification was performed as previously described using GoTaq qPCR Master Mix (Promega, Madison, USA) and oligonucleotide primers specific for 16S rDNA or eubacteria or strictly *Pseudomonas* OTU750018 (S8 Table). Samples were equilibrated to the actin of *H*. *oligactis* and to the overall bacterial load of each polyp by Eub-primers.

### Phylogenetic analysis

To identify the phylogenetic relations of *Pseudomonas* and spirochetes bacteria, we compared their 16S rRNA sequences to previously published sets [22,84]. Sequence alignment was generated using Clustal_x [85] and the phylogenetic tree was inferred using MEGA 6 software [86]. A model-test was used to estimate the best fit substitution models for phylogenetic analyses. For the maximum-likelihood analyses, genes were tested using the General Time Reversible (GTR + I) model. A bootstrap test with 1000 replicated for maximum likelihood and random seed was conducted.

### Bacteria motility assay

In order to test the motility of *T*. *parva* DSM21527 and *Pseudomonas* OTU750018, we inoculated 40,000 bacterial cells in 2 μl culture medium into semi-solid agar plates (3.5% Agar; Difco Leptospira Medium) and incubated at 29°C for 72 h. Bacteria cell number was counted using a Neubauer chamber and adjusted to 20,000 bacteria per μl. In order to test the interaction between *T*. *parva* DSM21527 and *Pseudomonas* OTU750018, the bacteria were inoculated either onto the same spot or 1 cm apart into semi-solid agar plates and incubated as described above.

### Transmission electron microscopy

To visualize bacteria in the mesoglea of Hydra at high resolution, we used transmission electron microscopy (TEM) analyssis of thin sections of healthy and tumorous *H*. *oligactis* polyps. The sections were prepared following the previously published protocol [87]. TEM was done using Tecnai G^2^ Spirit/BioTWIN (FEI Company, Thermo Fisher Scientific, USA).

### Immunohistochemistry

Immunohistochemical detection of female germline marker, the periculin protein, in whole mount *Hydra* preparations was performed as described [24] using a polyclonal mouse antiserum against periculin 1a protein (1:500 diluted produced by Bosch lab) and Alexa488 conjugated donkey–anti-mouse secondary antibodies (2 μg/ml; Invitrogen, USA). Phalloidin staining was conducted as described previously [88]. Detection of *Pseudomonas* and *T*. *parva* on the mesoglea was performed using SYBR-gold staining. Isolated mesoglea fragments were rinsed in sterile ddH_2_O and stained with SYBR-gold (1:20,000 in ddH_2_O; Thermo Fisher Scientific) for 3 min in the dark at room temperature. After a brief washing, in ddH_2_O, the mesoglea was embedded in Moviol-Dabco [89]. Confocal microscopy images were taken using TCS SP1 laser-scanning confocal microscope (Leica, Germany).

### Mesoglea thickness measurement

To measure the thickness of the mesoglea in healthy and tumorous polyps, individual hydras were relaxed in 2% urethane and fixed with 4% formaldehyde, dehydrated in ethanol and embedded into LR-White resin (Ted Pella, Redding, CA, USA) according to the manufacturer’s instruction. Semi-thin sections (0.5 μm thick) were cut using Ultracut S ultratome, mounted on slides and stained with methylene blue/azur II as described previously [1]. Average thickness of mesoglea was measured on 10 random locations of each section, and twelve sections for each condition (control and tumor) were evaluated.

### Cell number quantification using flow cytometry

To estimate the number of epithelial cells per polyp, we disintegrated healthy control and tumorous hydras and subjected to flow cytometry analysis following previously established protocol [27]. For each replicate, individual polyps were digested in 100 μl of 50 U/ml Pronase E (Serva) in an isotonic culture medium for 4 h at 18 °C. Living cells were counted on BD FACSCalibur with CellQuestPro v5.2 (Becton–Dickinson) using forward scatter and side scatter parameters. Gating and further analyses were performed with FCSalyzer 0.9.13-alpha (https://sourceforge.net/projects/fcsalyzer/).

### Fitness assay

To assess the growth rate of healthy and tumorous *H*. *oligactis* polyps, single polyps served as founder for a clonal population. Progeny of this polyp and all other following offspring’s were placed individually into wells of 12-well plate until and fed daily ad libitum. The number of polyps per well was recorded daily until at least 100 polyps were generated. For population growth rate calculation the experiment was split in sub experiments at the second generation. Since *Hydra* grows clonally and the genotype is the same for one line, the resulting sub-experiments were treated as independent replicates. For growth rate calculation a regression of the log^2^-transformed polyp number per time unit was performed and the slope of the curve as descriptor for growth rate was used. For data analysis a MySQL-database as well as custom written python and R functions were utilized.

### Transplantation experiments

In order to introduce *Pseudomonas* into tumor-free *H*. *oligactis* polyps we performed lateral transplantation experiment. Fragments of the middle body column were excised from healthy *H*. *oligactis* polyps, antibiotic-treated polyps devoid of *Pseudomonas*, or from tumor-free buds. The tissue fragments were transplanted onto tumor-free *H*. *oligactis* polyps as previously described [1]. In contrast to the original protocol, the grafted tissue fragment was removed 12 h after the transplantation in order to avoid migration of the cells from the transplant into the recipient tissue. In a similar way, spirochetes from *H*. *circumcincta* were introduced into healthy *H*. *oligactis* polyps.

### Ethics statement

No ethical approval is required to perform experiments on the pre-bilaterian invertebrate *Hydra oligactis*.

## Supporting information

S1 Fig Presence of *Pseudomonas* and *Turneriella* on the mesoglea of *Hydra*. **(A)** Transmission electron microscopy images reveal the presence of *Pseudomonas* in the mesoglea (ECM) of healthy H. oligactis. Rod shaped *Pseudomonas* colonizes the mesoglea in high abundance and appears to dissolve the ECM in the surrounding (red arrows; scale bar: 1 μm). **(B)** Spirochetes colonize the ECM in tumorous *H*. *oligactis* polyps. Electron microscopy images of the spirochetes colonizing the ECM (scale bar: 1μm) characterized by a helical-coiled cross section (scale bar; 1 μm/500 nm).
(TIF)

S2 Fig Injection of *T. parva* results in tumor formation. Gradual acquisition of tumorous phenotype is observed within 50 days after injection of *T*. *parva* culture into healthy *H*. *oligactis* polyps. Image timeline of the same animal post *T*. *parva* injection. After around 30 days the tumor phenotype becomes conspicuous (scale bar: 5mm).
(TIF)

S3 Fig Low presence of *T. parva* in healthy polyps injected with spirochetes. While injection of *T*. *parva* into healthy *H*. *oligactis* polyps often results in tumor development, some polyps still manifest a healthy phenotype. Relative abundance of bacterial OTUs based on 16S rDNA sequencing and resolved on the order level illustrates that the abundance of *T*. *parva* in these injected polyps without a phenotype is below 1%.
(TIF)

S4 Fig Antibiotic treatment results in complete elimination of spirochetes from tumorous polyps and obliterates the tumor. **(A)** Electrophoretic analysis of PCR products amplified using the universal primers Eub27F and Eub1492R specific for Eubacteria 16S rDNA gene [46]. gDNA samples from non-treated tumor polyps (Tum., two replicates) were used as a positive control and show a clear amplification product of expected size 1400 bp, consistent with the presence of microbiota in these polyps. Absence of amplification products in the samples from antibiotics-treated polyps (Antib., two replicates) confirms their germ-free status. Sterile water sample (H_2_0) was used as a negative control. **(B)** Antibiotic treatment and subsequent feeding eliminates the tumor phenotype within 30 days (scale bar: 0.5 cm). The feeding of germ-free polyps with *Artemia* nauplii reintroduces a food-derived microbial community dominated by the Sphingobacteriales and Burkholderiales bacteria. Importantly, members of Leptospirales order are absent from these recovered polyps. **(C)** Antibiotic treatment of tumorous polyps generated by injection of *T*. *parva* into healthy *H*. *oligactis* polyps similarly resulted in complete tumor eradication. 16rDNA of *T*. *parva* can not be amplified from these recovered polyps (inset) indicating the absence of spirochetes.
(TIF)

S5 Fig Tumor growth is accompanied by morphological and developmental alterations. **(A)** Thickness of the mesoglea increases significantly in tumor polyps compared to control polyps (*n = 12*). **(B)** Epithelial cell number doubles in tumorous polyps compared to control polyps (*n* = 6). ***—p<0.001.
(TIF)

S6 Fig Gradual acquisition of the tumor-specific phenotype in polyps injected with *T. parva*. **(A)** Number of tentacles per polyp gradually increases after injection of healthy polyps with *T*. *parva* and reaches the values characteristic for tumorous polyps 120 days post injection. **(B)** The progressive development of the tumorous phenotype is accompanied by a gradual increase in the density of *T*. *parva* in the polyps. 40 days post injection the density of *T*. *parva* in *Hydra* tissue is over 500-fold higher than shortly after injection. ***—p<0.001.
(TIF)

S7 Fig Intraindividual variation in microbiota composition among healthy control and tumorous polyps. The relative bacterial abundance is deduced from 16S rDNA sequencing and resolved at the phylum level. Six replicates, of healthy polyps (Control, c1-6) and six tumor-bearing polyps (Tumor, t1-6) were analyzed. Averaged values are represented on Fig 1A and 1C.
(TIF)

S8 Fig *T. parva* is not able to settle onto the antibiotic-treated polyps. Electrophoretic analysis of PCR products amplified using the specific primers for *T*. *parva* 16S rDNA as well as the primers specific for Eubacteria 16S rDNA gene [46]. Absence of *T*. *parva* 16S rDNA amplification products (left, six replicates) indicates that the injected spirochetes are not able to colonize the antibiotics-treated polyps. A clear band is amplified from gDNA of tumorous polyps (tum) harboring the spirochetes. Sterile water sample (H_2_0) was used as a negative control. Amplification of Eubacteria 16S rDNA (six replicates) is a result of presence of other microbes (S4B Fig) introduced by feeding.
(TIF)

S9 Fig Introduction of *Pseudomonas* into the mesoglea densely populated by *Turneriella* via tissue grafting consistently induces tumors. Electrophoretic analysis of PCR products amplified using the specific primers for *Pseudomonas* and *Turneriella* 16S rDNA from randomly selected tumorous (t) and healthy (h) polyps (each two replicates) resulting from the grafting experiment presented on Fig 4C and 4D. All tested tumor bearing polyps had both, spirochetes and *Pseudomonas* in their microbiome. In all polyps, where transplantation did not result in tumor formation, *Pseudomonas* was missing.
(TIF)

S10 Fig *H. circumcincta* harbors spirochetes in the mesoglea that are capable to induce tumors in *H. oligactis*. **(A)** *H*. *circumcincta* microbiome is dominated by spirochetes (data from Franzenburg et al., PNAS 2013), however tumors have never been detected in this *Hydra* species. Notably, bacteria of Pseudomonadales order are virtually absent from *H*. *circumcincta*. **(B)** Confocal microscopy confirms abundant spirochetes in the mesoglea of *H*. *circumcincta*. No rod-shaped pseudomonas cells can be detected in mesoglea of this Hydra species. **(C)** Phylogenetic analysis of 16S rDNA gene sequence identifies the spirochete from *H*. *circumcincta* as members of Leptospirales family, yet very distant from the Turneriella OTU4017244 and *T*. *parva* used in our study. Neighbour-joining phylogram with numbers at nodes representing bootstrap support values calculated by 1000 iterations. **(D)** Introduction of *Leptospira biflexa* spirochetes from *H*. *cricumcincta* into healthy *H*. *oligactis* polyps that harbor *Pseudomonas* OTU750018 in mesoglea results in tumor formation in 20% cases. Amplification of Leptospira 16S rDNA fragment (insert) confirms successful colonization of the recipient polyps.
(TIF)

S11 Fig The flagellum assembly system encoded in the genomes of *Pseudomonas* OTU750018 and *T. parva* DSM21527. **(A)** Almost complete repertoire of genes coding for proteins commonly involved in the assembly of bacterial flagellum are present in the *Pseudomonas* genome (green boxes). Few genes are either absent or not discovered by our annotation pipeline (white boxes). **(B)** *T*. *parva* genome also encodes multiple genes of the flagellum machinery.
(TIF)

S12 Fig The bacterial secretion systems encoded in the genomes of *Pseudomonas* OTU750018 and *T. parva* DSM21527. **(A)** Genes coding for the bacterial secretion systems II & VI are present in the Pseudomonas genome and shaded in green. Additionaly, multiple genes coding for Sec-SRP machinery are found in Pseudomonas genome. Components of other secretion systems (Type III, IV and V) are missing in the genome of *Pseudomonas* (white). **(B)** Genes linked to the bacterial secretion systems II are partial present in the *T*. *parva* genome and shaded in green. Genes coding for components of other secretions systems (white boxes) were not detected in *T*. *parva* genome.
(TIF)

S13 Fig Repertoire of ABC-transporters encoded in the genomes of *Pseudomonas* OTU750018 and *T. parva* DSM21527. **(A)** Multiple genes coding for different ABC-transporters and present in the *Pseudomonas* genome (shaded in green). **(B)** Relatively few genes encoding different ABC transporters are present in the *T*. *parva* genome (shaded in green). Most of these ABC-transporter complexes appear incomplete and thus are likely not functional in *T*. *parva*.
(TIF)

S14 Fig The genome of *Pseudomonas* OTU750018 carries a complete prophage sequence. Entire complex of bacteriophage genes are annotated in a single cluster within *Pseudomonas* genome.
(TIF)

S15 Fig *Pseudomonas* OTU750018 produces outer membrane vesicles. TEM images of *Pseudomonas* in the mesoglea of *Hydra* reveal outer membrane vesicles (OMVs) on the surface of every of *Pseudomonas* cell (red arrows; scale bar: 50/100/200 nm).
(TIF)

S16 Fig Motility of *Pseudomonas* OTU750018 and *T. parva* DSM21527. **(A)** *Pseudomonas* colony spreads on semi-liquid agar plate as a symmetric circle. The motility zone (M) was calculated as an average of two diameters of a colony measured perpendicular to each other. **(B)** *T*. *parva* colonies also spread as perfectly round circles. **(C)** If both bacteria are inoculated on the same spot, they keep high motility as spread as symmetric circles. **(D)** Quantification of the bacteria motility using the motility zone measurement on the second (day 2) and third day (day 3) after inoculation. Both bacteria plated together (*Pseudomonas* +*T* and *T*. *parva +P*) show significantly higher motility compared to both bacteria plated alone (*Pseudomonas*–*T* and *T*. *parva*–*S*), indicating that the motility of the both, *Pseudomonas* OTU750018 and *T*. *parva* DSM21527, is activated in the presence of the second bacterium. This points to an interaction that takes place between two bacteria. ***—p<0.001
(TIF)

S17 Fig Motility of *T. parva* DSM21527 increases in the presence of living *Pseudomonas* OTU750018 cells. **(A)** If *T*. *parva* and *Pseudomonas* are inoculated onto the same plate on a distance 1 cm and monitored over 72 h, a prominent asymmetric spread of the *T*. *parva* colony is observed. The colony protrudes towards the *Pseudomonas* colony (red arrow). **(B)** In order to quantify this asymmetric motility, we used a motility bias parameter Δ as a difference between two diameters of *T*. *parva* colony measured perpendicular to each other, with the second (d2) being located on the line of Pseudomonas colony. **(C)** *T*. *parva* demonstrates a clear positive motility bias only in the presence of living *Pseusomonas* cells (+*Pseud*.). Cell-free supernatant of *Pseudomonas* culture (+*Pseud*. supernat.) does not cause motility bias in *T*. *parva*. *n* = 10, ***—p<0.00.1 **(D)** A sterile-filtered supernatant from *Pseudomonas* culture does not alter the motility of *T*. *parva*. **(E)** No asymmetry in motility is observed if two *T*. *parva* colonies are inoculated and grow next to each other for 48 hours. **(F)** 72h after inoculation, both *T*. *parva* colonies merge.
(TIF)

S18 Fig *T. parva* DSM21527 and *Pseudomonas* OTU750018 demonstrate different motility patterns. **(A)** Frames selected from the S1 Movie demonstrate position of four *T*. *parva* cells (T1-T4). As S1 Movie clearly shows, the movement of *T*. *parva* is relatively slow (note the time stamp) and not directed, schematically represented on the right panel. **(B)** Frames selected from the S2 Movie demonstrate position of two Pseudomonas cells (P1 and P2). The swimming of *Pseudomonas* is fast (note the time stamp and S2 Movie) and directed, schematically represented on the right panel.
(TIF)

S19 Fig Interaction between *T. parva* DSM21527 and *Pseudomonas* OTU750018. In the presence of *Pseudomonas*, *T*. *parva* demonstrates a behavioral shift and moves directly towards *Pseudomonas* and establishes a contact. **(A)** Frames selected from S3 Movie demonstrate position of one *T*. *parva* cell (T) and one Pseudomonas cell (P). Red arrow indicates direction of *T*. *parva* swimming, red arrow indicates the point of contact between *T*. *parva* and *Pseudomonas*. As seen on S3 Movie, *T*. *parva* detaches from *Pseudomonas* after a brief contact and swims in opposite direction (right panel). **(B)** Frames selected from S4 Movie demonstrate a recurrent contact between *T*. *parva* (T) and *Pseudomonas* (P) accompanied by a circular *T*. *parva* swimming around the *Pseudomonas* cell. **(C)** Frames selected from S5 Movie demonstrate a stable contact between one *T*. *parva* and two *Pseudomonas* cells resulting in a joint swimming of the bacteria. **(D)** Frames selected from S6 Movie demonstrate a stable contact between one *T*. *parva* and two *Pseudomonas* cells resulting in a joint swimming of the bacteria.
(TIF)

S1 Table Accession numbers of 16S rDNA sequences used for phylogenetic analysis of *Pseudomonas* and spirochetes. (XLSX)

S2 Table Annotation of genes in the genome of *Pseudomonas* OTU750018 isolate. (XLSX)

S3 Table Putative secondary metabolites produced by *Pseudomonas* deduced by ANTI-SMASH analysis of Pseudomonas OTU750018 genome. (XLS)

S4 Table Annotated phage genes detected in the genome sequence of *Pseudomonas* OTU750018 and identified using PHAST tool. (XLSX)

S5 Table Annotation of genes in the *T. parva* DSM21527 genome. (XLSX)

S6 Table Putative secondary metabolites produced by the spirochetes deduced by ANTI-SMASH analysis of *T. parva* DSM21527 genome. (XLSX)

S7 Table Top50 genes of *Pseudomonas* and *Turneriella* differentially expressed in the tumor. Genes validated in qRT-PCR are highlighted green. (XLSX)

S8 Table Oligonicleotide primers used to amplify bacterial 16S rDNA genes and virulence gene in qRT-PCR. (XLSX)

S1 Data Full genome sequence of *Pseudomonas* OTU750018 isolate. (ZIP)

S2 Data Full genome sequence of *T. parva* DSM21527. (ZIP)

S1 Movie Motility of *T. parva* DSM21527. (AVI)

S2 Movie Motility of *Pseudomonas* OTU750018 isolate. (AVI)

S3 Movie Interaction of *T. parva* DSM21527 and *Pseudomonas* OTU750018: Directed swimming of *T. parva* towards Pseudomonas and transient contact. (AVI)

S4 Movie Interaction of *T. parva* DSM21527 and *Pseudomonas* OTU750018: Circular swimming of *T. parva* around *Pseudomonas* and recurrent contact. (AVI)

S5 Movie Interaction of *T. parva* DSM21527 and *Pseudomonas* OTU750018: Stable contact and aggregated swimming of *T. parva* around *Pseudomonas*. (AVI)

S6 Movie Interaction of *T. parva* DSM21527 and *Pseudomonas* OTU750018: Stable contact and aggregated swimming of *T. parva* around *Pseudomonas*. (AVI)
