# Supplementary figures and images for: Dynamic interactions within the host-associated microbiota cause tumor formation in the basal metazoan Hydra
Source: PLoS Pathog. 2020 Mar 19;16(3):e1008375. doi: 10.1371/journal.ppat.1008375 (PMC7081986; doi:10.1371/journal.ppat.1008375)

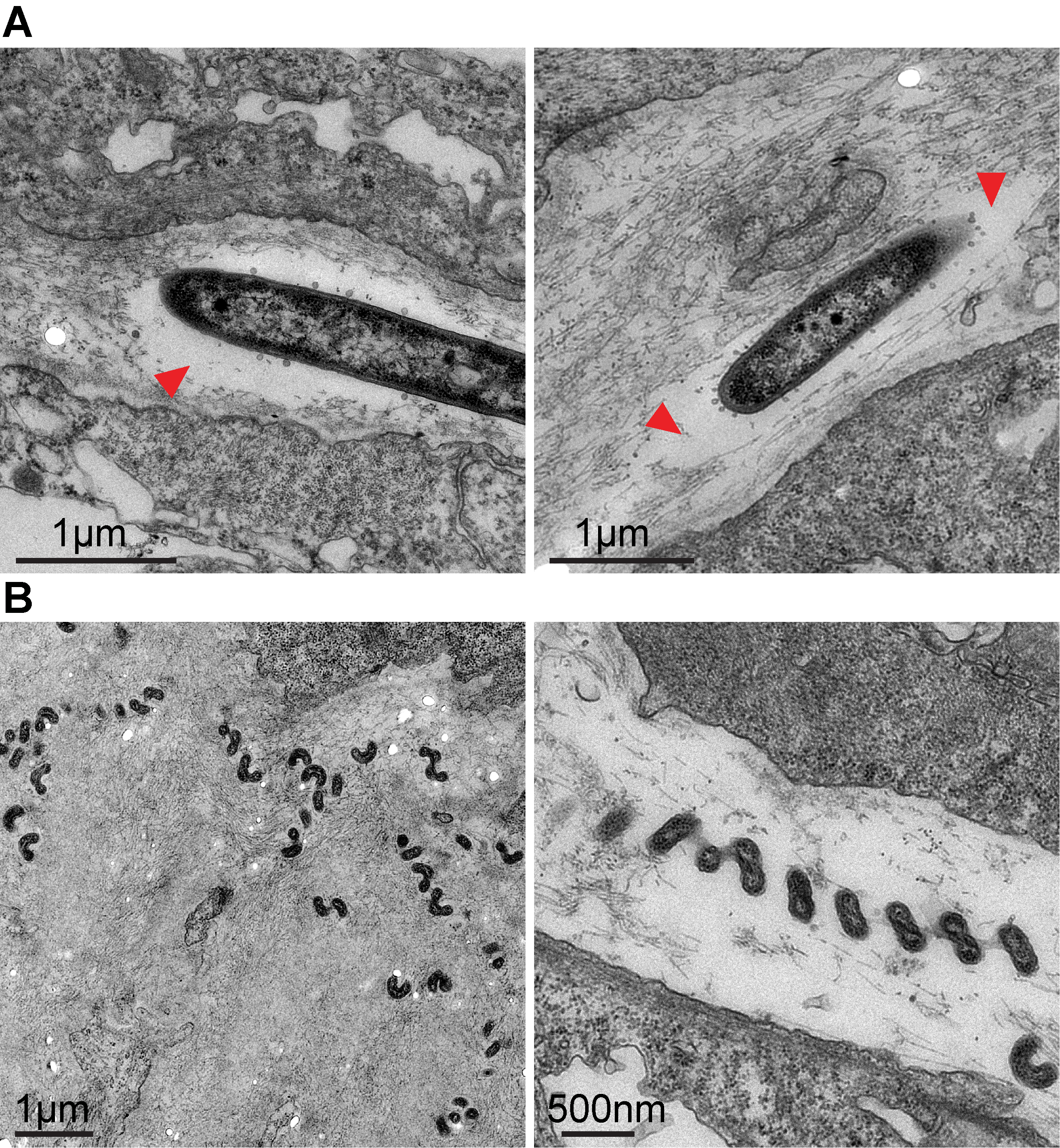

Supplement: S1 Fig — (A) Transmission electron microscopy images reveal the presence of Pseudomonas in the mesoglea (ECM) of healthy H. oligactis. Rod shaped Pseudomonas colonizes the mesoglea in high abundance and appears to dissolve the ECM in the surrounding (red arrows; scale bar: 1 μm). (B) Spirochetes colonize the ECM in tumorous H. oligactis polyps. Electron microscopy images of the spirochetes colonizing the ECM (scale bar: 1μm) characterized by a helical-coiled cross section (scale bar; 1 μm/500 nm). (TIF) [file ppat.1008375.s001.tif]

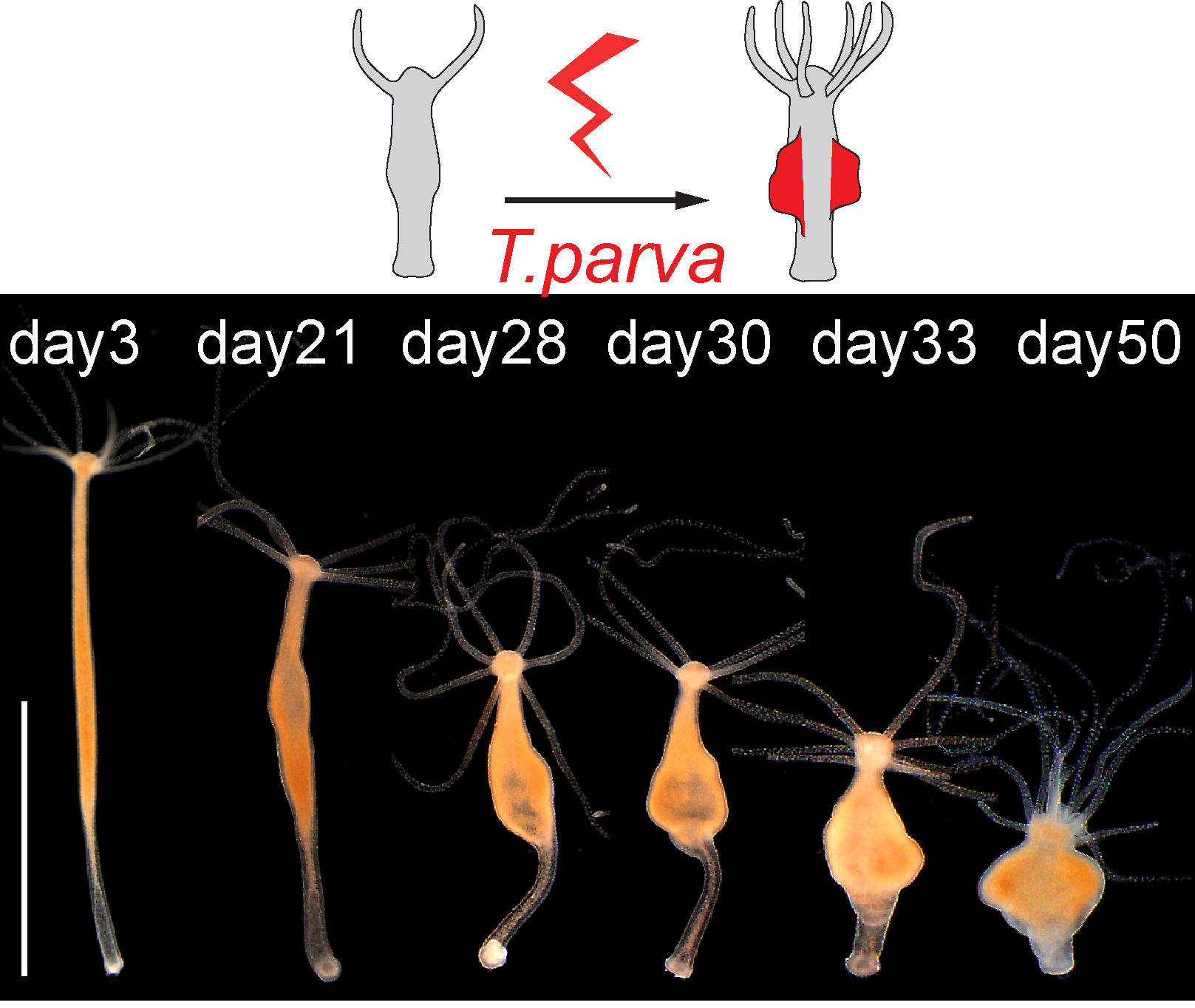

Supplement: S2 Fig — Gradual acquisition of tumorous phenotype is observed within 50 days after injection of T. parva culture into healthy H. oligactis polyps. Image timeline of the same animal post T. parva injection. After around 30 days the tumor phenotype becomes conspicuous (scale bar: 5mm). (TIF) [file ppat.1008375.s002.tif]

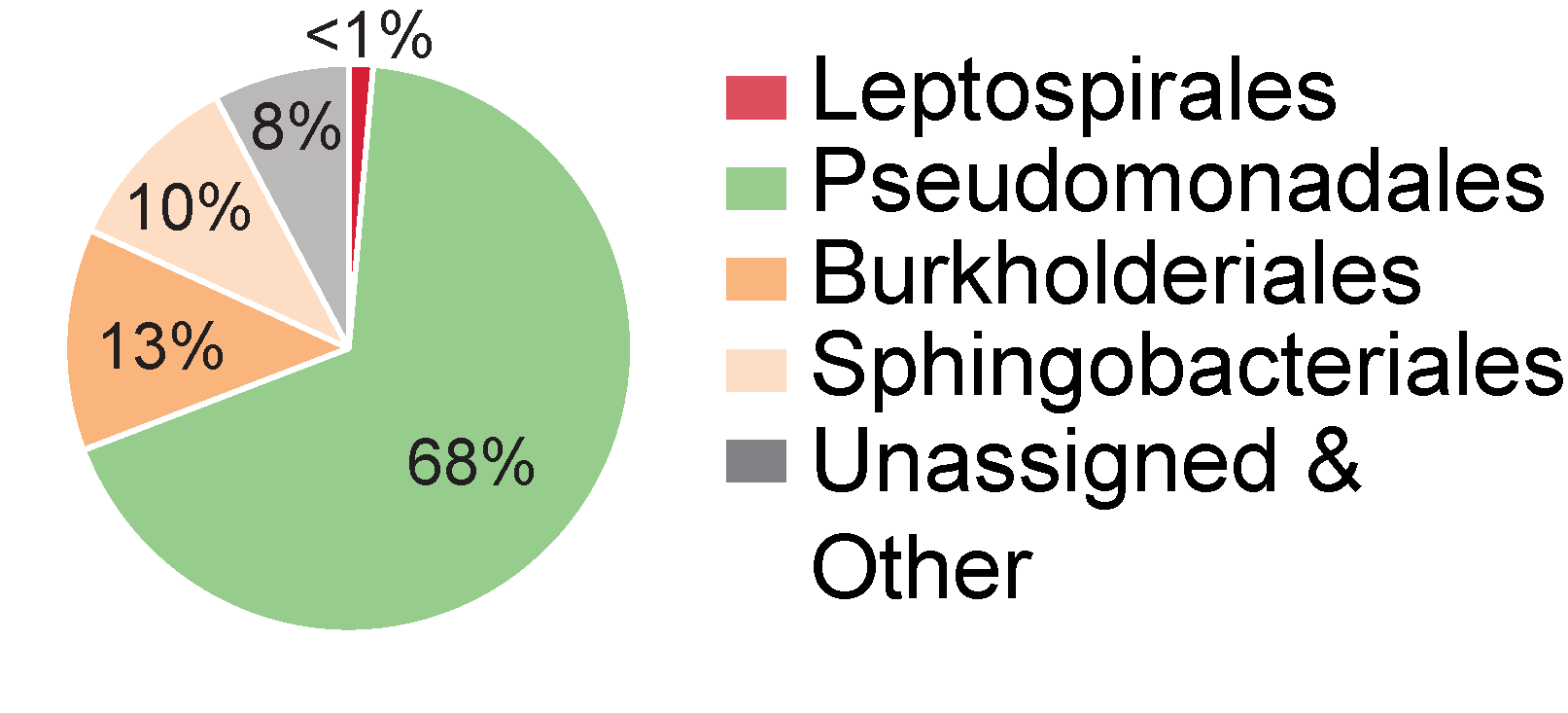

Supplement: S3 Fig — While injection of T. parva into healthy H. oligactis polyps often results in tumor development, some polyps still manifest a healthy phenotype. Relative abundance of bacterial OTUs based on 16S rDNA sequencing and resolved on the order level illustrates that the abundance of T. parva in these injected polyps without a phenotype is below 1%. (TIF) [file ppat.1008375.s003.tif]

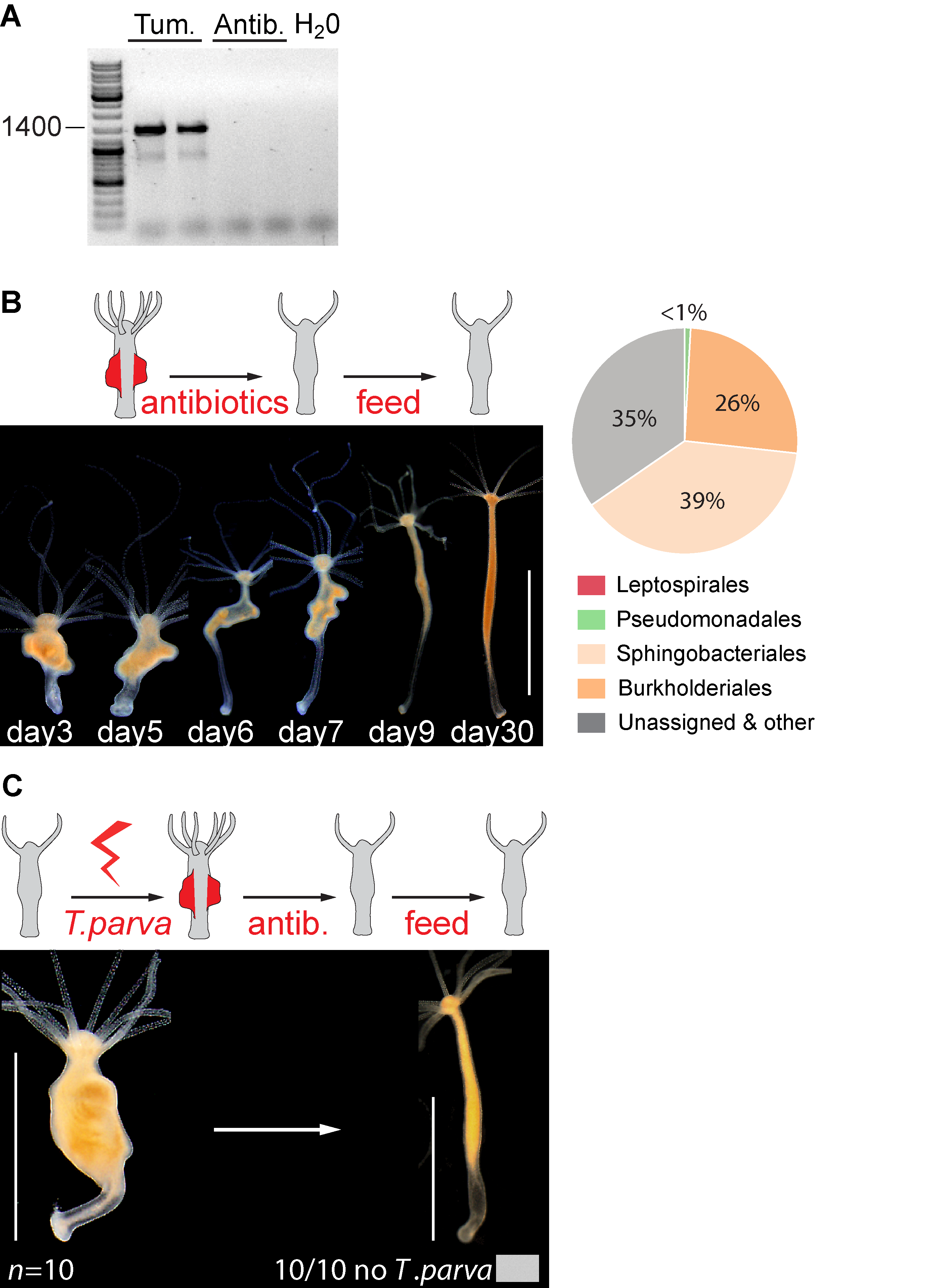

Supplement: S4 Fig — (A) Electrophoretic analysis of PCR products amplified using the universal primers Eub27F and Eub1492R specific for Eubacteria 16S rDNA gene [46]. gDNA samples from non-treated tumor polyps (Tum., two replicates) were used as a positive control and show a clear amplification product of expected size 1400 bp, consistent with the presence of microbiota in these polyps. Absence of amplification products in the samples from antibiotics-treated polyps (Antib., two replicates) confirms their germ-free status. Sterile water sample (H20) was used as a negative control. (B) Antibiotic treatment and subsequent feeding eliminates the tumor phenotype within 30 days (scale bar: 0.5 cm). The feeding of germ-free polyps with Artemia nauplii reintroduces a food-derived microbial community dominated by the Sphingobacteriales and Burkholderiales bacteria. Importantly, members of Leptospirales order are absent from these recovered polyps. (C) Antibiotic treatment of tumorous polyps generated by injection of T. parva into healthy H. oligactis polyps similarly resulted in complete tumor eradication. 16rDNA of T. parva can not be amplified from these recovered polyps (inset) indicating the absence of spirochetes. (TIF) [file ppat.1008375.s004.tif]

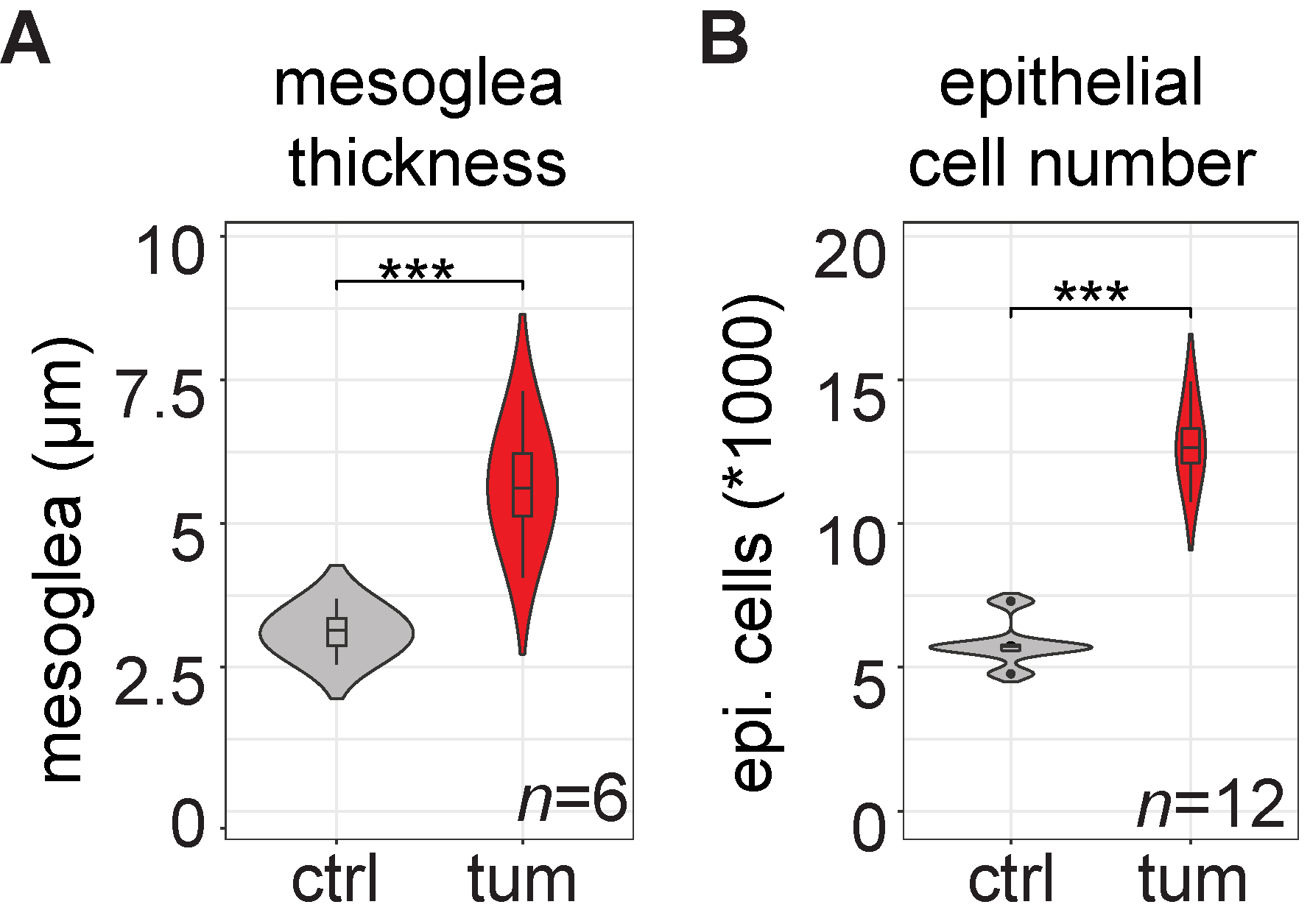

Supplement: S5 Fig — (A) Thickness of the mesoglea increases significantly in tumor polyps compared to control polyps (n = 12). (B) Epithelial cell number doubles in tumorous polyps compared to control polyps (n = 6). ***—p<0.001. (TIF) [file ppat.1008375.s005.tif]

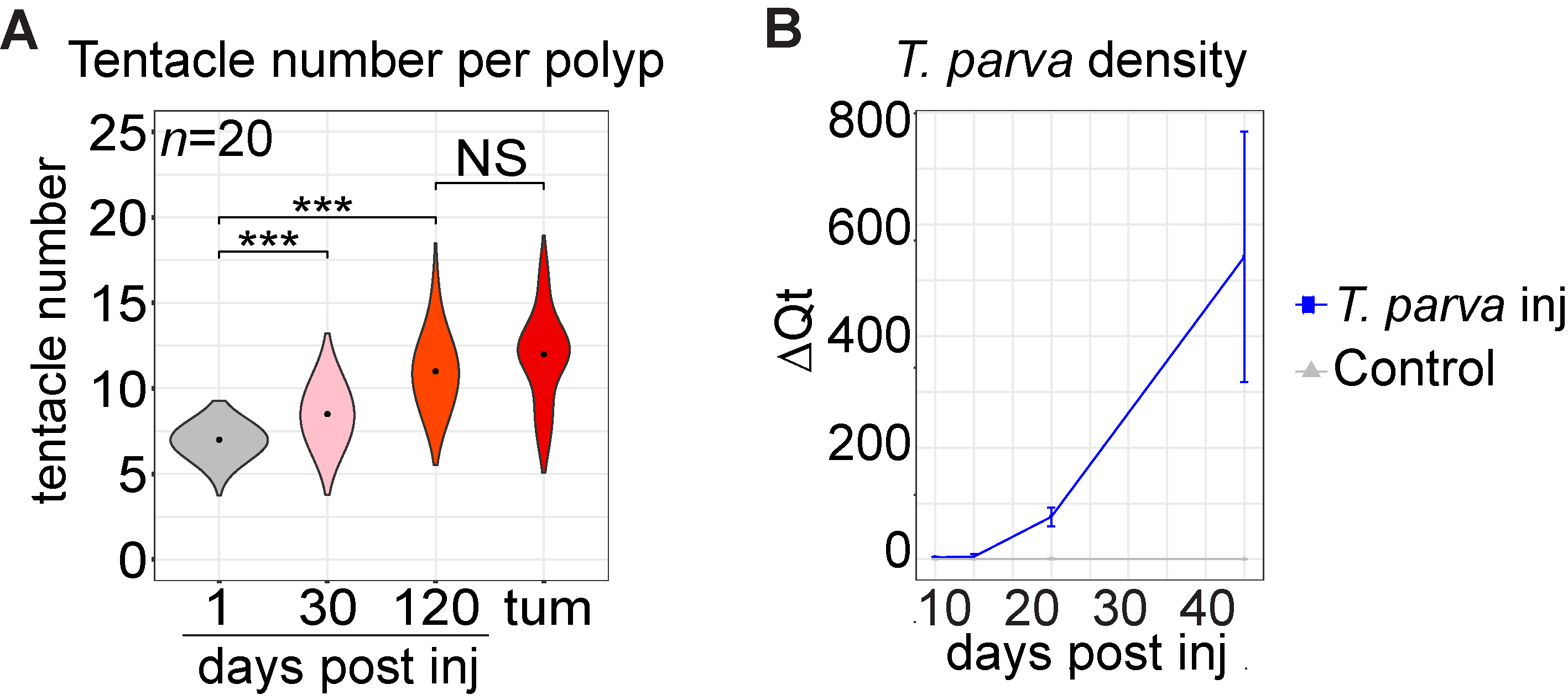

Supplement: S6 Fig — (A) Number of tentacles per polyp gradually increases after injection of healthy polyps with T. parva and reaches the values characteristic for tumorous polyps 120 days post injection. (B) The progressive development of the tumorous phenotype is accompanied by a gradual increase in the density of T. parva in the polyps. 40 days post injection the density of T. parva in Hydra tissue is over 500-fold higher than shortly after injection. ***—p<0.001. (TIF) [file ppat.1008375.s006.tif]

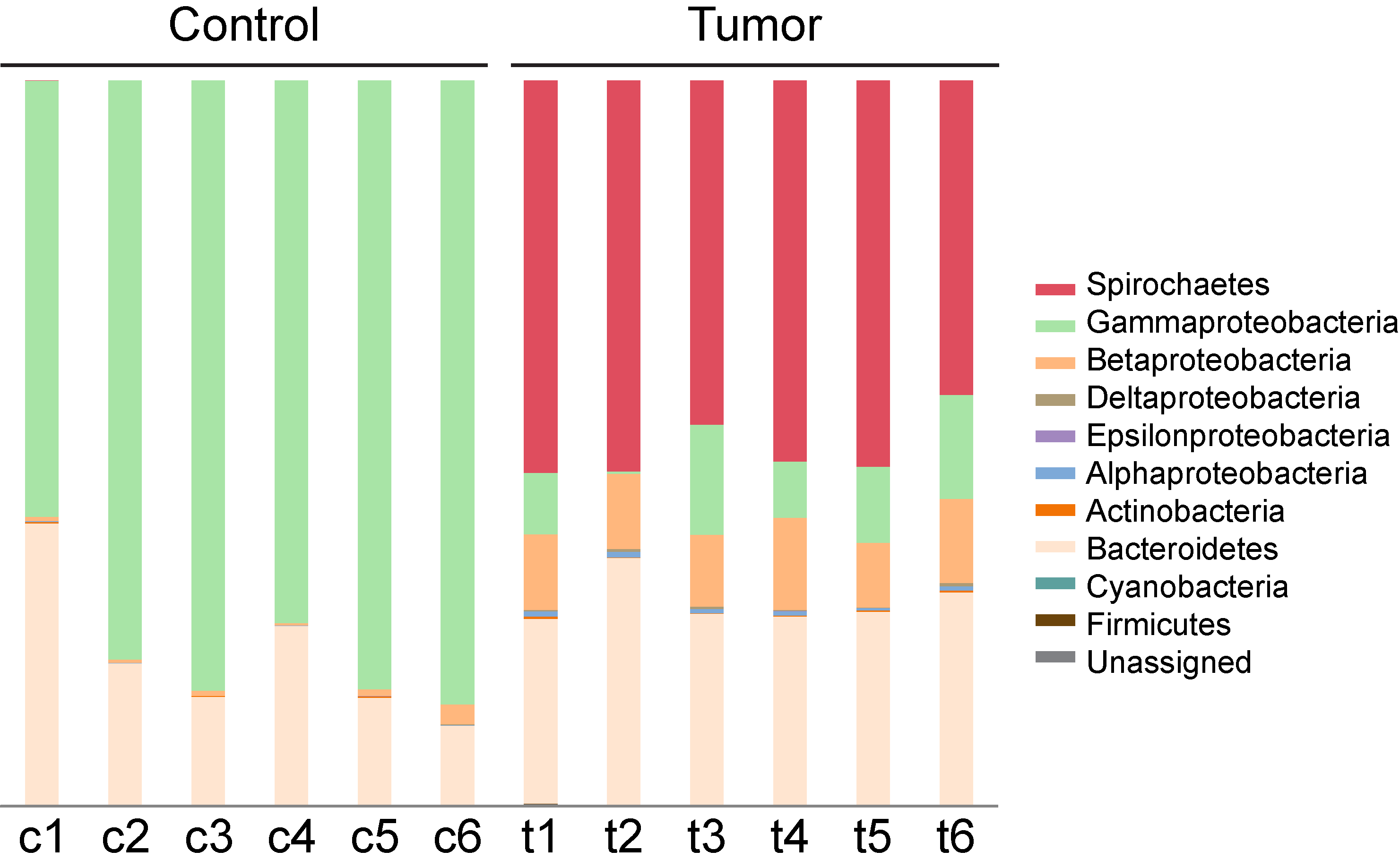

Supplement: S7 Fig — The relative bacterial abundance is deduced from 16S rDNA sequencing and resolved at the phylum level. Six replicates, of healthy polyps (Control, c1-6) and six tumor-bearing polyps (Tumor, t1-6) were analyzed. Averaged values are represented on Fig 1A and 1C. (TIF) [file ppat.1008375.s007.tif]

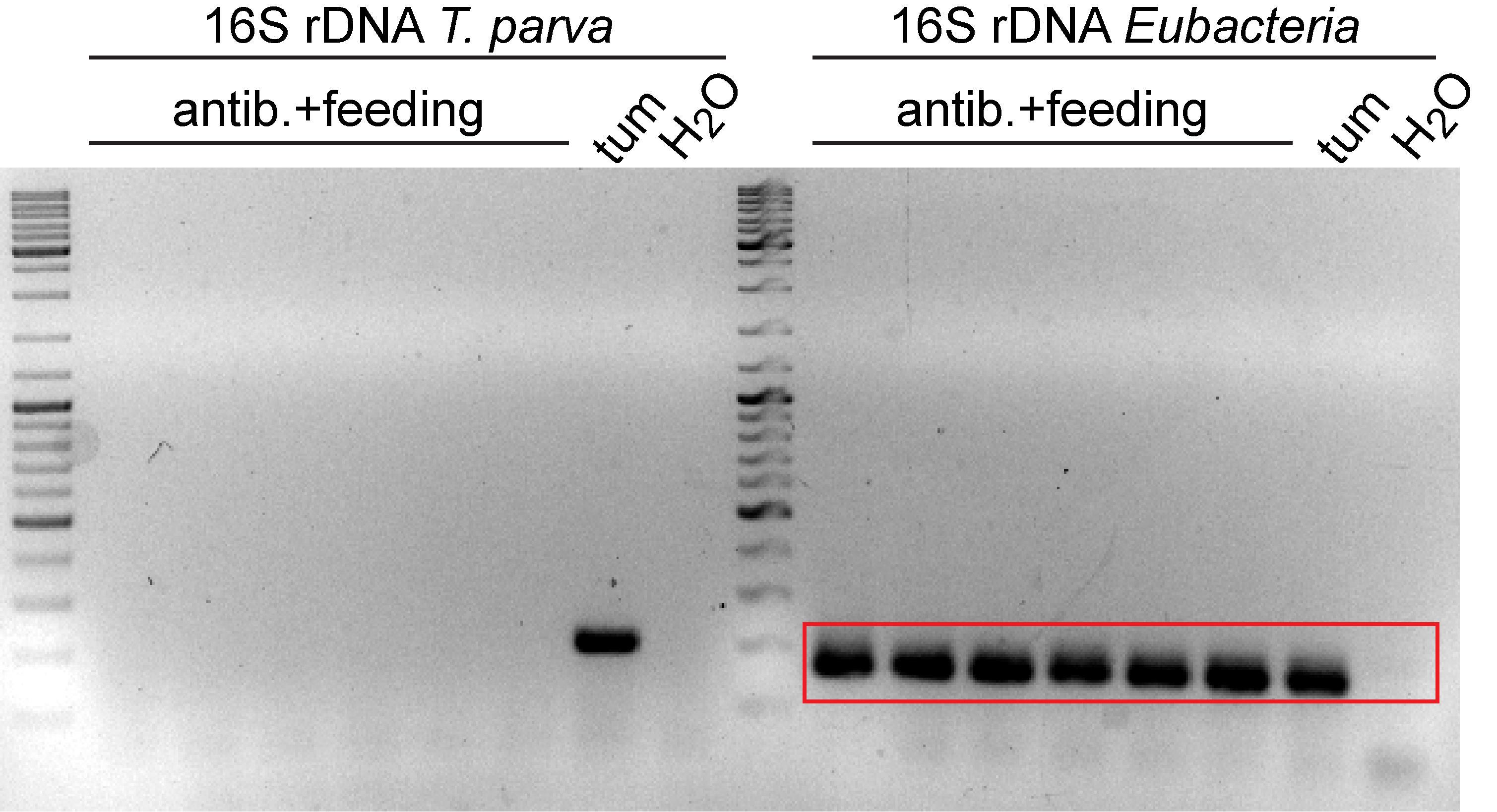

Supplement: S8 Fig — Electrophoretic analysis of PCR products amplified using the specific primers for T. parva 16S rDNA as well as the primers specific for Eubacteria 16S rDNA gene [46]. Absence of T. parva 16S rDNA amplification products (left, six replicates) indicates that the injected spirochetes are not able to colonize the antibiotics-treated polyps. A clear band is amplified from gDNA of tumorous polyps (tum) harboring the spirochetes. Sterile water sample (H20) was used as a negative control. Amplification of Eubacteria 16S rDNA (six replicates) is a result of presence of other microbes (S4B Fig) introduced by feeding. (TIF) [file ppat.1008375.s008.tif]

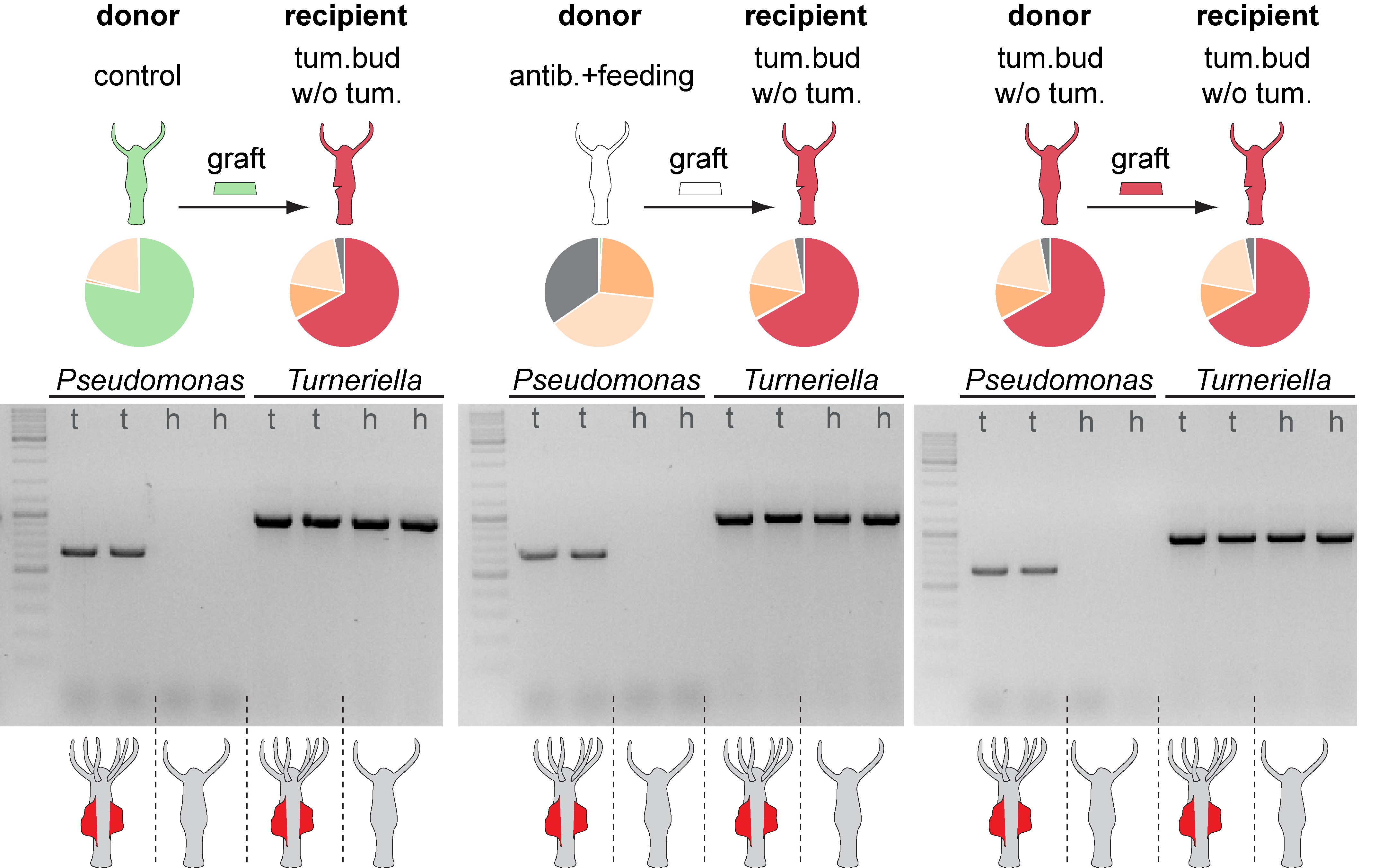

Supplement: S9 Fig — Electrophoretic analysis of PCR products amplified using the specific primers for Pseudomonas and Turneriella 16S rDNA from randomly selected tumorous (t) and healthy (h) polyps (each two replicates) resulting from the grafting experiment presented on Fig 4C and 4D. All tested tumor bearing polyps had both, spirochetes and Pseudomonas in their microbiome. In all polyps, where transplantation did not result in tumor formation, Pseudomonas was missing. (TIF) [file ppat.1008375.s009.tif]

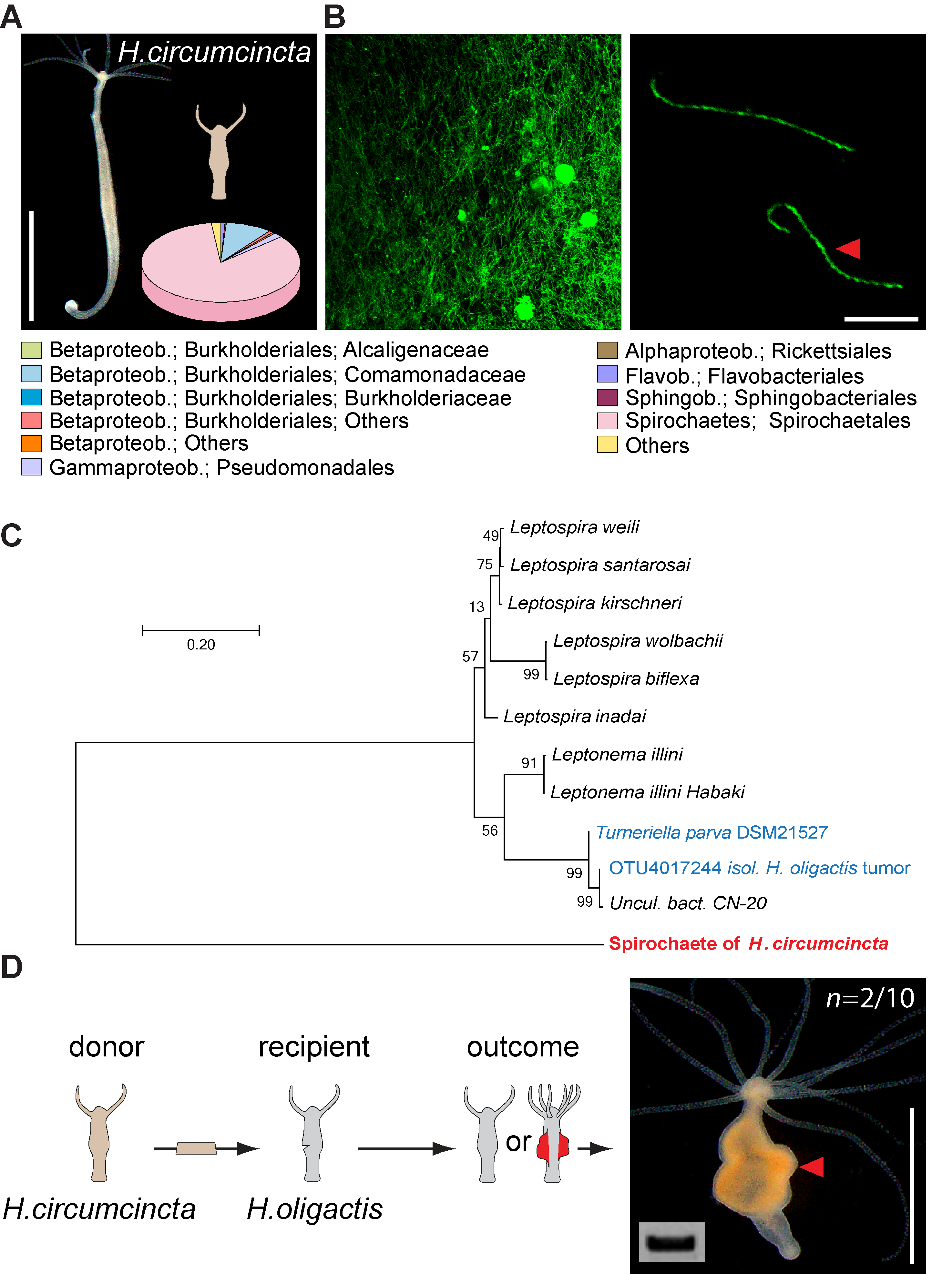

Supplement: S10 Fig — (A) H. circumcincta microbiome is dominated by spirochetes (data from Franzenburg et al., PNAS 2013), however tumors have never been detected in this Hydra species. Notably, bacteria of Pseudomonadales order are virtually absent from H. circumcincta. (B) Confocal microscopy confirms abundant spirochetes in the mesoglea of H. circumcincta. No rod-shaped pseudomonas cells can be detected in mesoglea of this Hydra species. (C) Phylogenetic analysis of 16S rDNA gene sequence identifies the spirochete from H. circumcincta as members of Leptospirales family, yet very distant from the Turneriella OTU4017244 and T. parva used in our study. Neighbour-joining phylogram with numbers at nodes representing bootstrap support values calculated by 1000 iterations. (D) Introduction of Leptospira biflexa spirochetes from H. cricumcincta into healthy H. oligactis polyps that harbor Pseudomonas OTU750018 in mesoglea results in tumor formation in 20% cases. Amplification of Leptospira 16S rDNA fragment (insert) confirms successful colonization of the recipient polyps. (TIF) [file ppat.1008375.s010.tif]

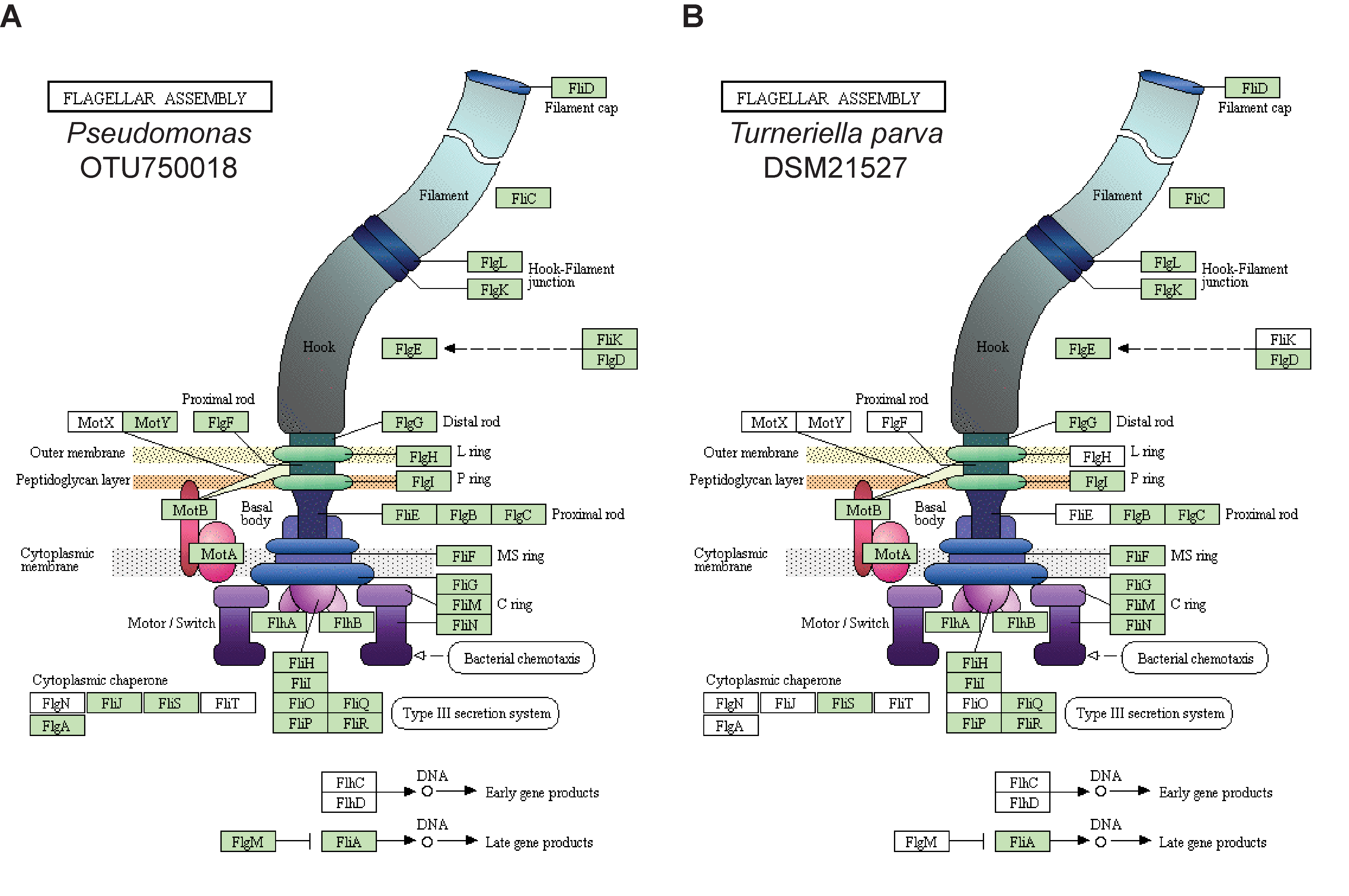

Supplement: S11 Fig — (A) Almost complete repertoire of genes coding for proteins commonly involved in the assembly of bacterial flagellum are present in the Pseudomonas genome (green boxes). Few genes are either absent or not discovered by our annotation pipeline (white boxes). (B) T. parva genome also encodes multiple genes of the flagellum machinery. (TIF) [file ppat.1008375.s011.tif]

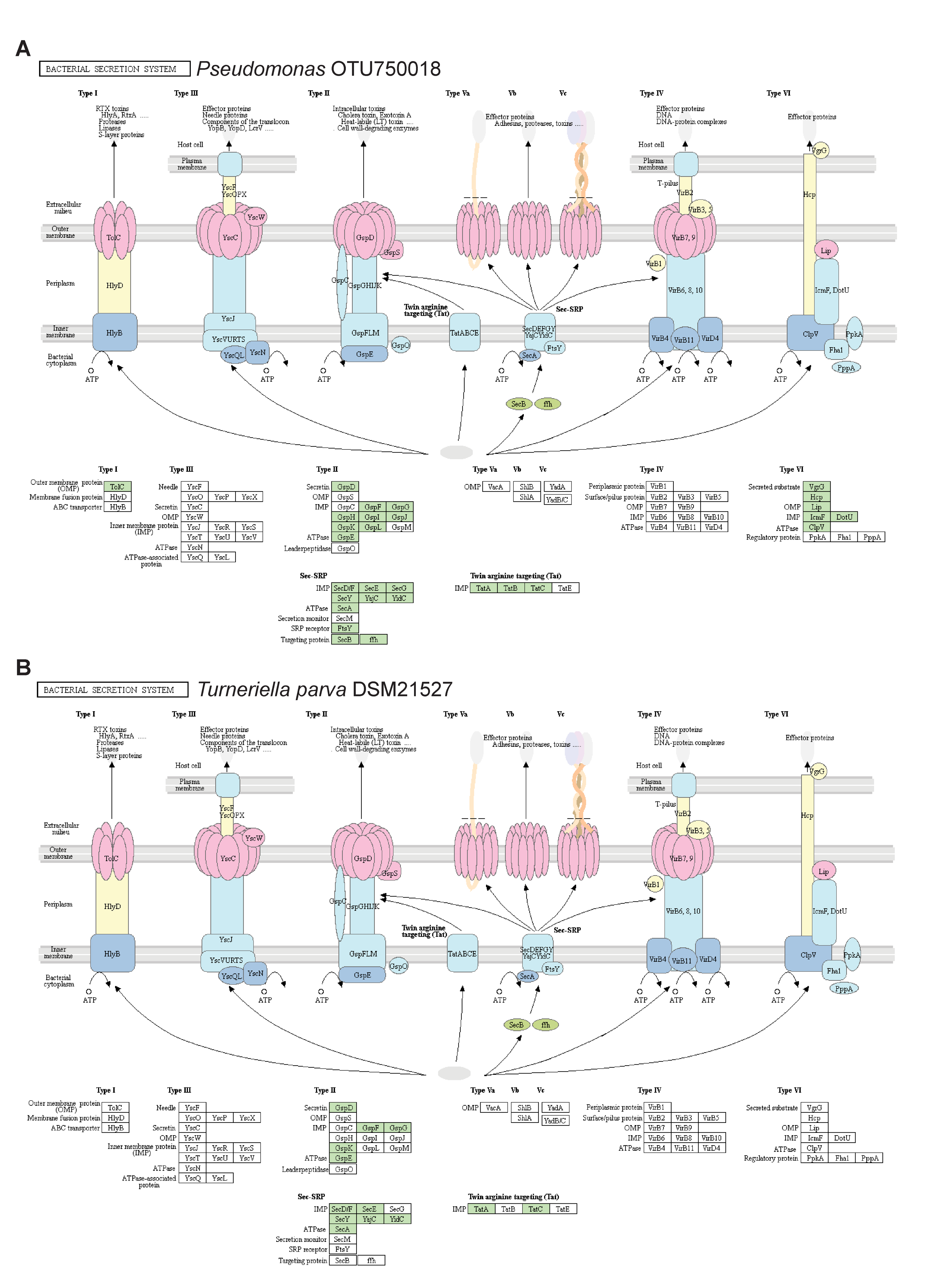

Supplement: S12 Fig — (A) Genes coding for the bacterial secretion systems II & VI are present in the Pseudomonas genome and shaded in green. Additionaly, multiple genes coding for Sec-SRP machinery are found in Pseudomonas genome. Components of other secretion systems (Type III, IV and V) are missing in the genome of Pseudomonas (white). (B) Genes linked to the bacterial secretion systems II are partial present in the T. parva genome and shaded in green. Genes coding for components of other secretions systems (white boxes) were not detected in T. parva genome. (TIF) [file ppat.1008375.s012.tif]

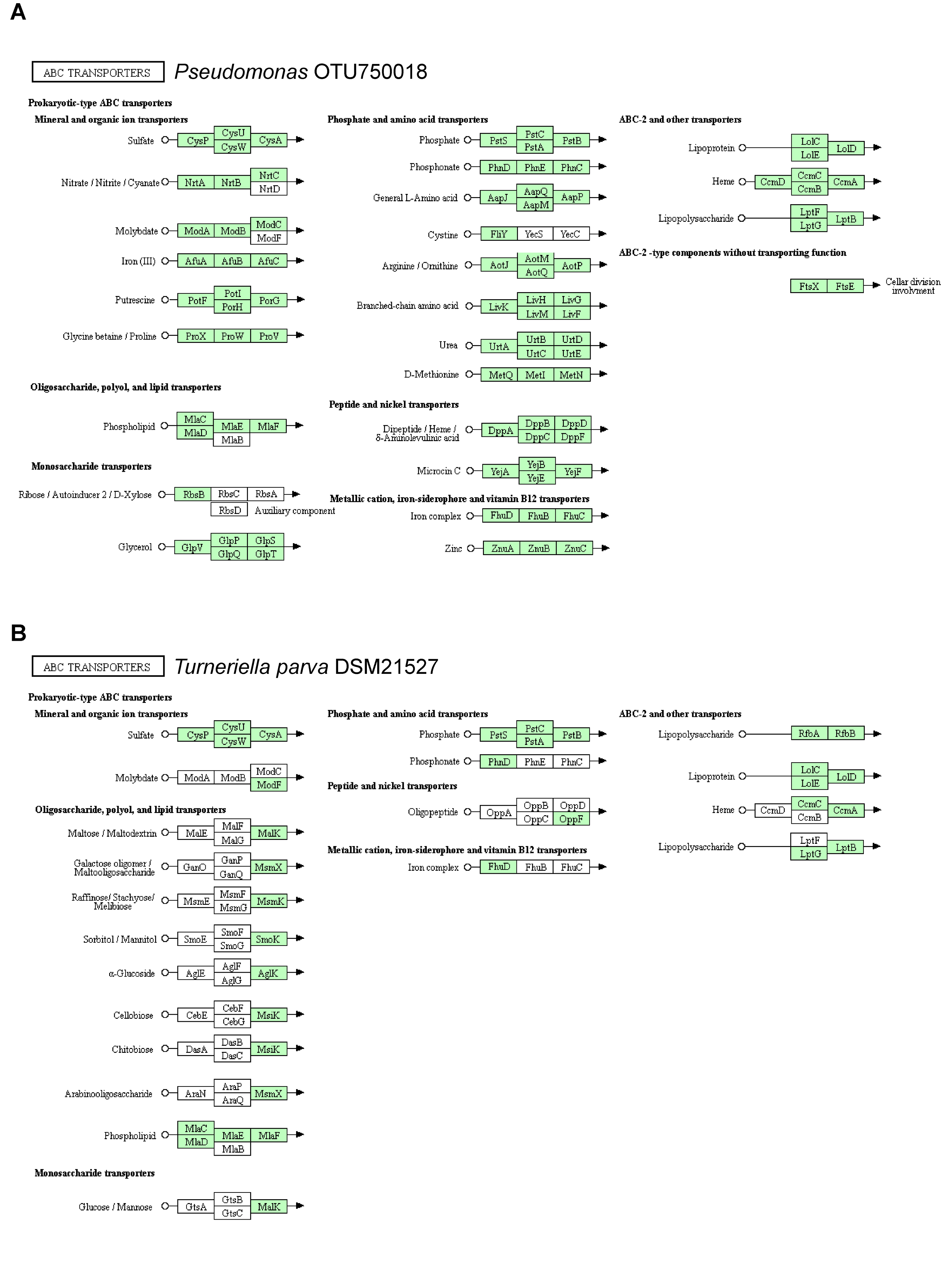

Supplement: S13 Fig — (A) Multiple genes coding for different ABC-transporters and present in the Pseudomonas genome (shaded in green). (B) Relatively few genes encoding different ABC transporters are present in the T. parva genome (shaded in green). Most of these ABC-transporter complexes appear incomplete and thus are likely not functional in T. parva. (TIF) [file ppat.1008375.s013.tif]

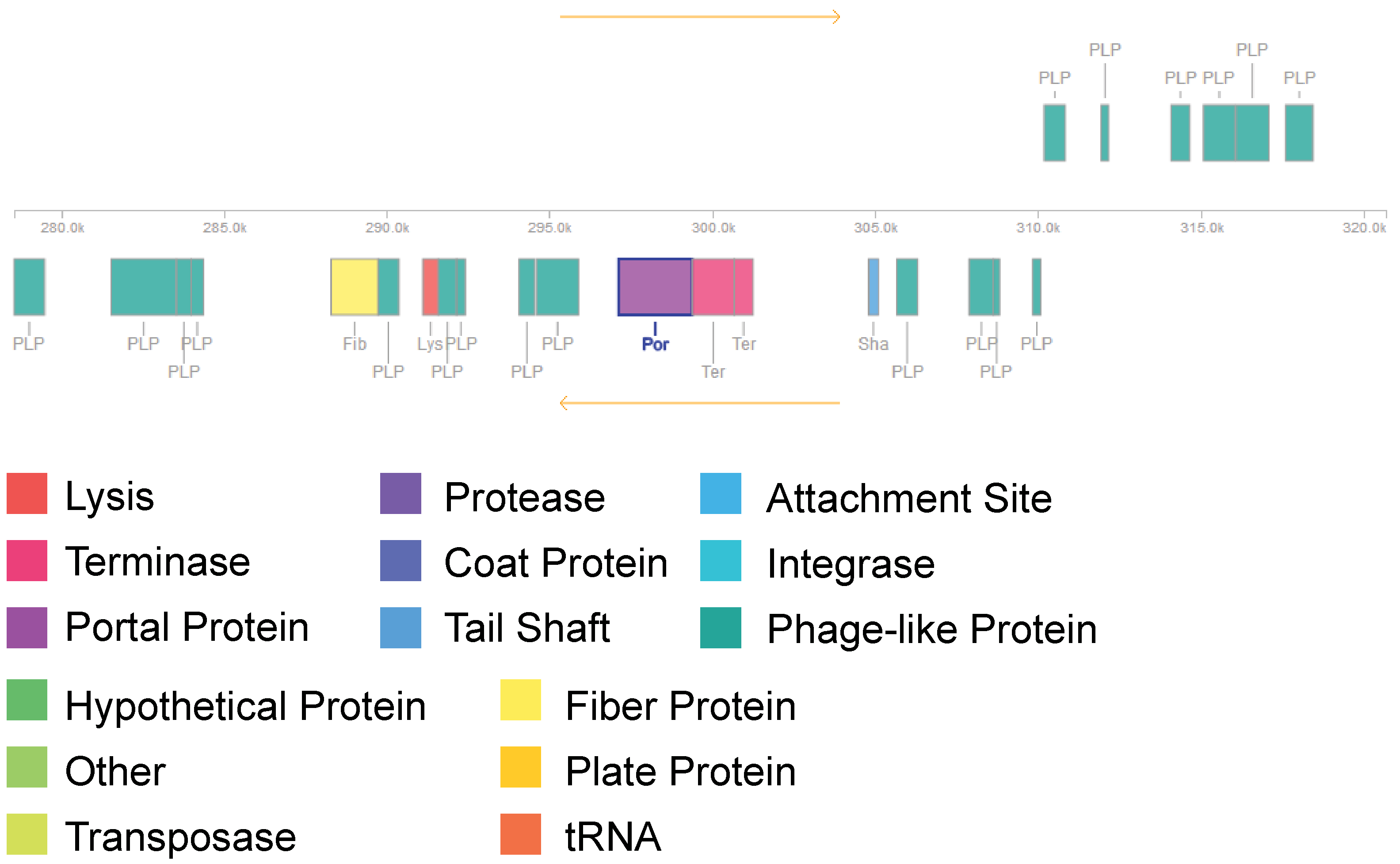

Supplement: S14 Fig — Entire complex of bacteriophage genes are annotated in a single cluster within Pseudomonas genome. (TIF) [file ppat.1008375.s014.tif]

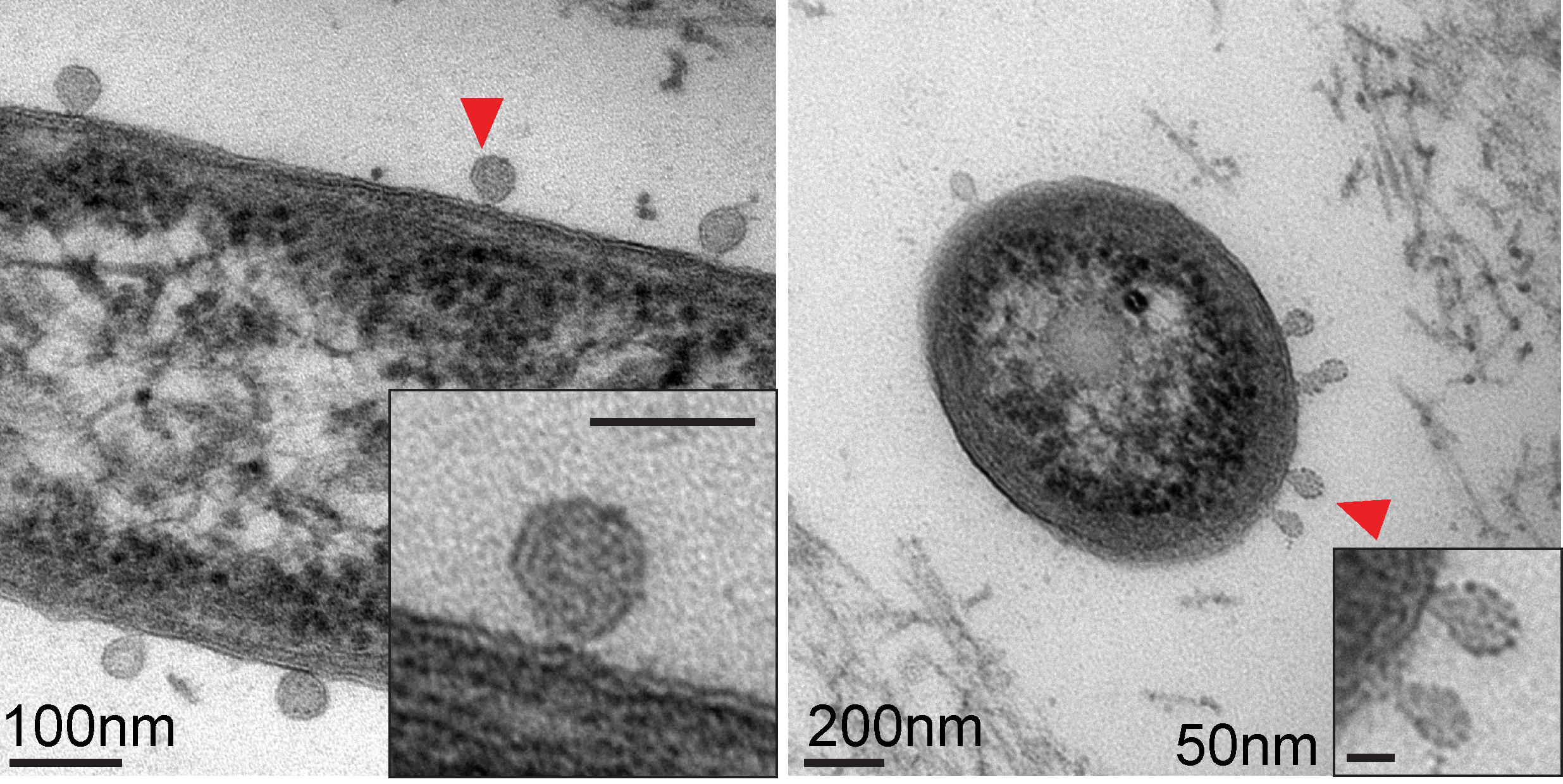

Supplement: S15 Fig — TEM images of Pseudomonas in the mesoglea of Hydra reveal outer membrane vesicles (OMVs) on the surface of every of Pseudomonas cell (red arrows; scale bar: 50/100/200 nm). (TIF) [file ppat.1008375.s015.tif]

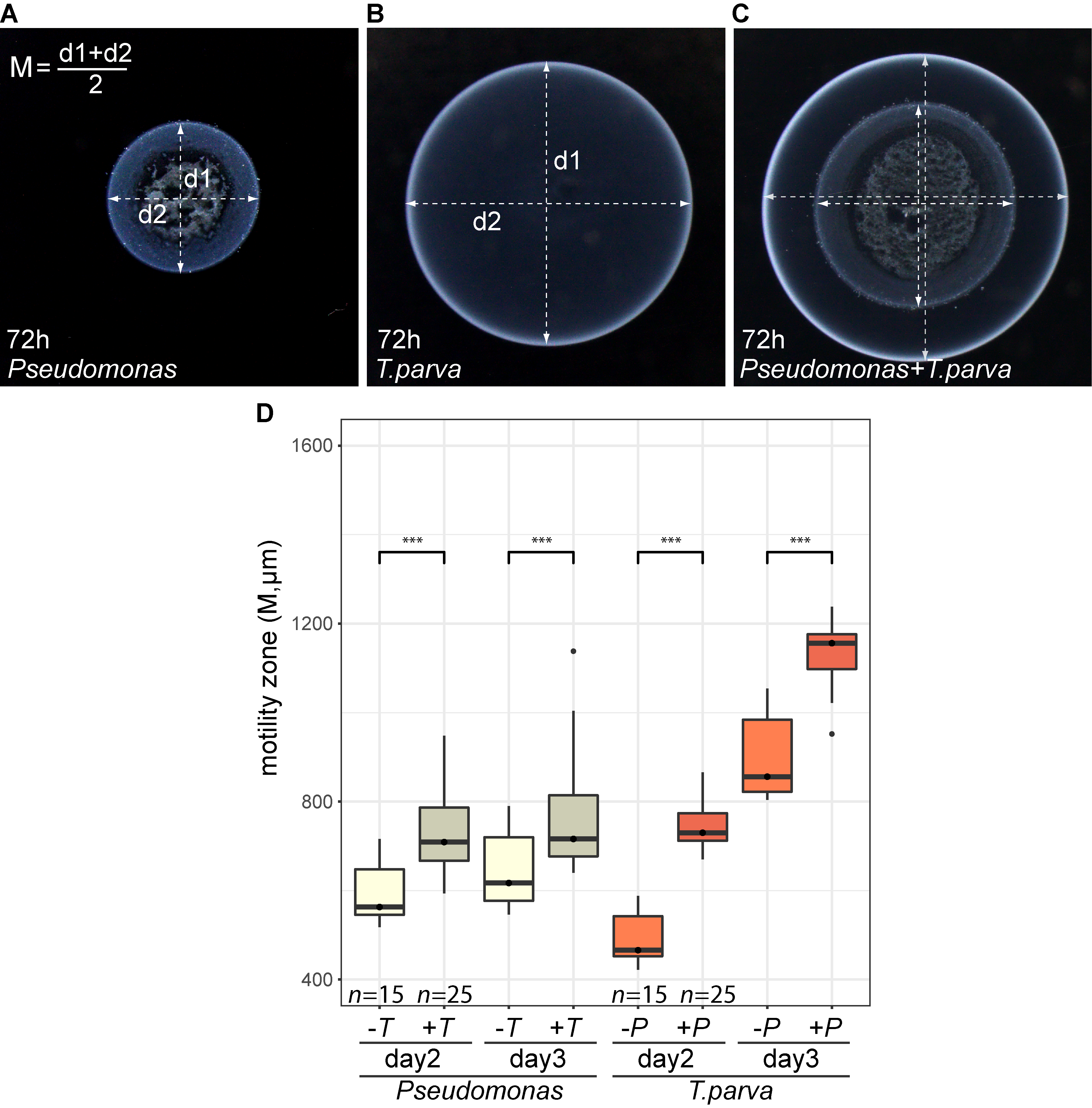

Supplement: S16 Fig — (A) Pseudomonas colony spreads on semi-liquid agar plate as a symmetric circle. The motility zone (M) was calculated as an average of two diameters of a colony measured perpendicular to each other. (B) T. parva colonies also spread as perfectly round circles. (C) If both bacteria are inoculated on the same spot, they keep high motility as spread as symmetric circles. (D) Quantification of the bacteria motility using the motility zone measurement on the second (day 2) and third day (day 3) after inoculation. Both bacteria plated together (Pseudomonas +T and T. parva +P) show significantly higher motility compared to both bacteria plated alone (Pseudomonas–T and T. parva–S), indicating that the motility of the both, Pseudomonas OTU750018 and T. parva DSM21527, is activated in the presence of the second bacterium. This points to an interaction that takes place between two bacteria. ***—p<0.001 (TIF) [file ppat.1008375.s016.tif]

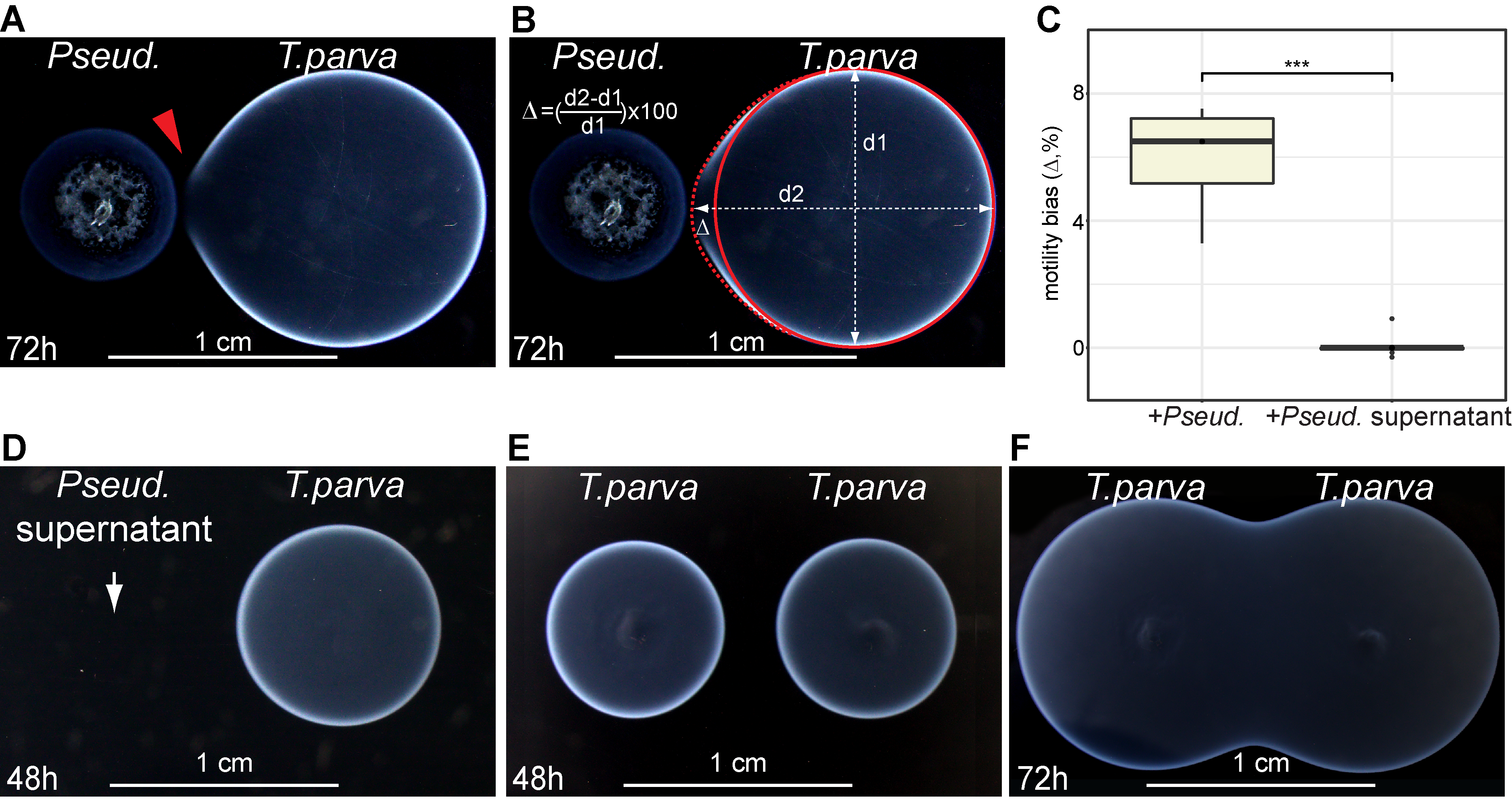

Supplement: S17 Fig — (A) If T. parva and Pseudomonas are inoculated onto the same plate on a distance 1 cm and monitored over 72 h, a prominent asymmetric spread of the T. parva colony is observed. The colony protrudes towards the Pseudomonas colony (red arrow). (B) In order to quantify this asymmetric motility, we used a motility bias parameter Δ as a difference between two diameters of T. parva colony measured perpendicular to each other, with the second (d2) being located on the line of Pseudomonas colony. (C) T. parva demonstrates a clear positive motility bias only in the presence of living Pseusomonas cells (+Pseud.). Cell-free supernatant of Pseudomonas culture (+Pseud. supernat.) does not cause motility bias in T. parva. n = 10, ***—p<0.00.1 (D) A sterile-filtered supernatant from Pseudomonas culture does not alter the motility of T. parva. (E) No asymmetry in motility is observed if two T. parva colonies are inoculated and grow next to each other for 48 hours. (F) 72h after inoculation, both T. parva colonies merge. (TIF) [file ppat.1008375.s017.tif]

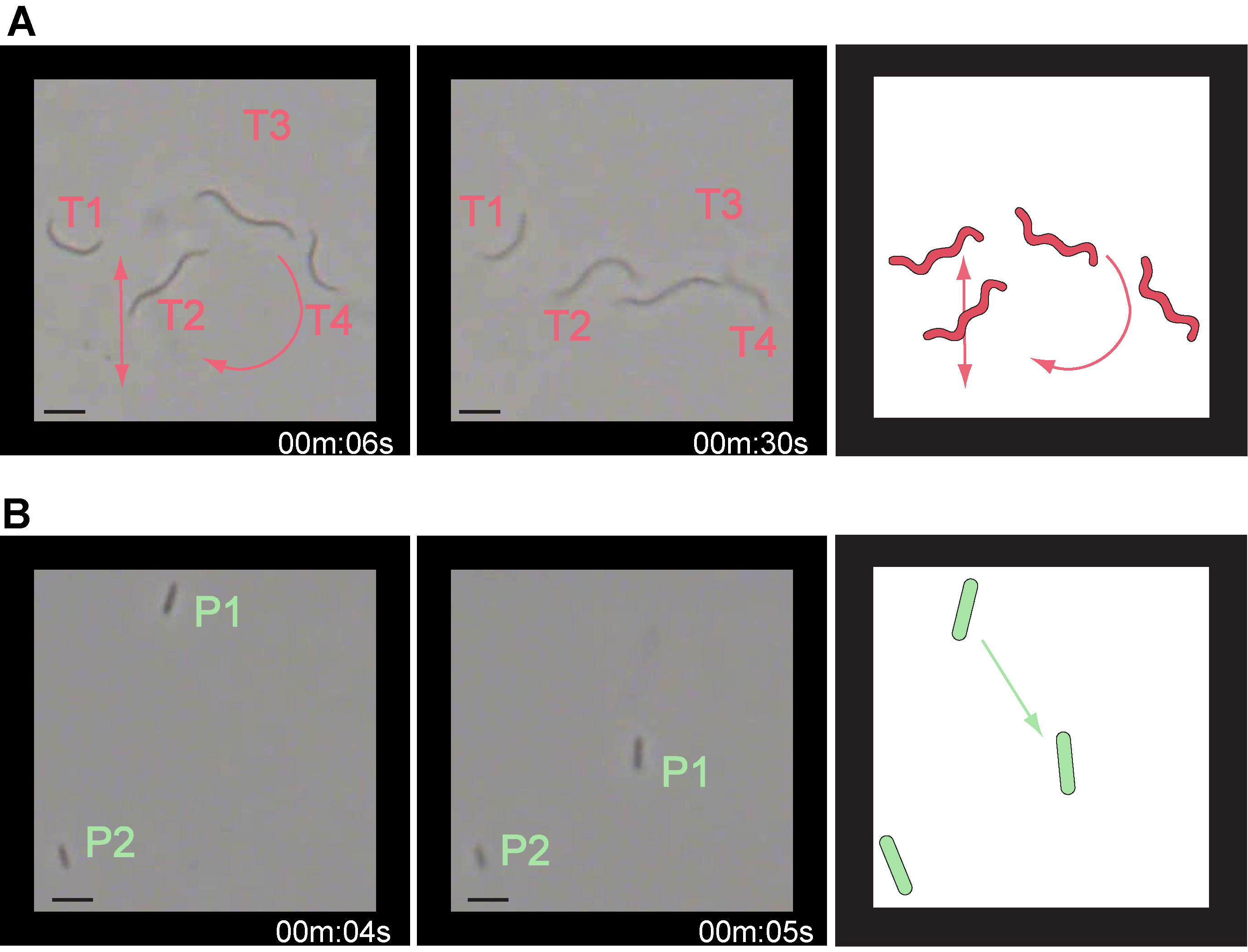

Supplement: S18 Fig — (A) Frames selected from the S1 Movie demonstrate position of four T. parva cells (T1-T4). As S1 Movie clearly shows, the movement of T. parva is relatively slow (note the time stamp) and not directed, schematically represented on the right panel. (B) Frames selected from the S2 Movie demonstrate position of two Pseudomonas cells (P1 and P2). The swimming of Pseudomonas is fast (note the time stamp and S2 Movie) and directed, schematically represented on the right panel. (TIF) [file ppat.1008375.s018.tif]

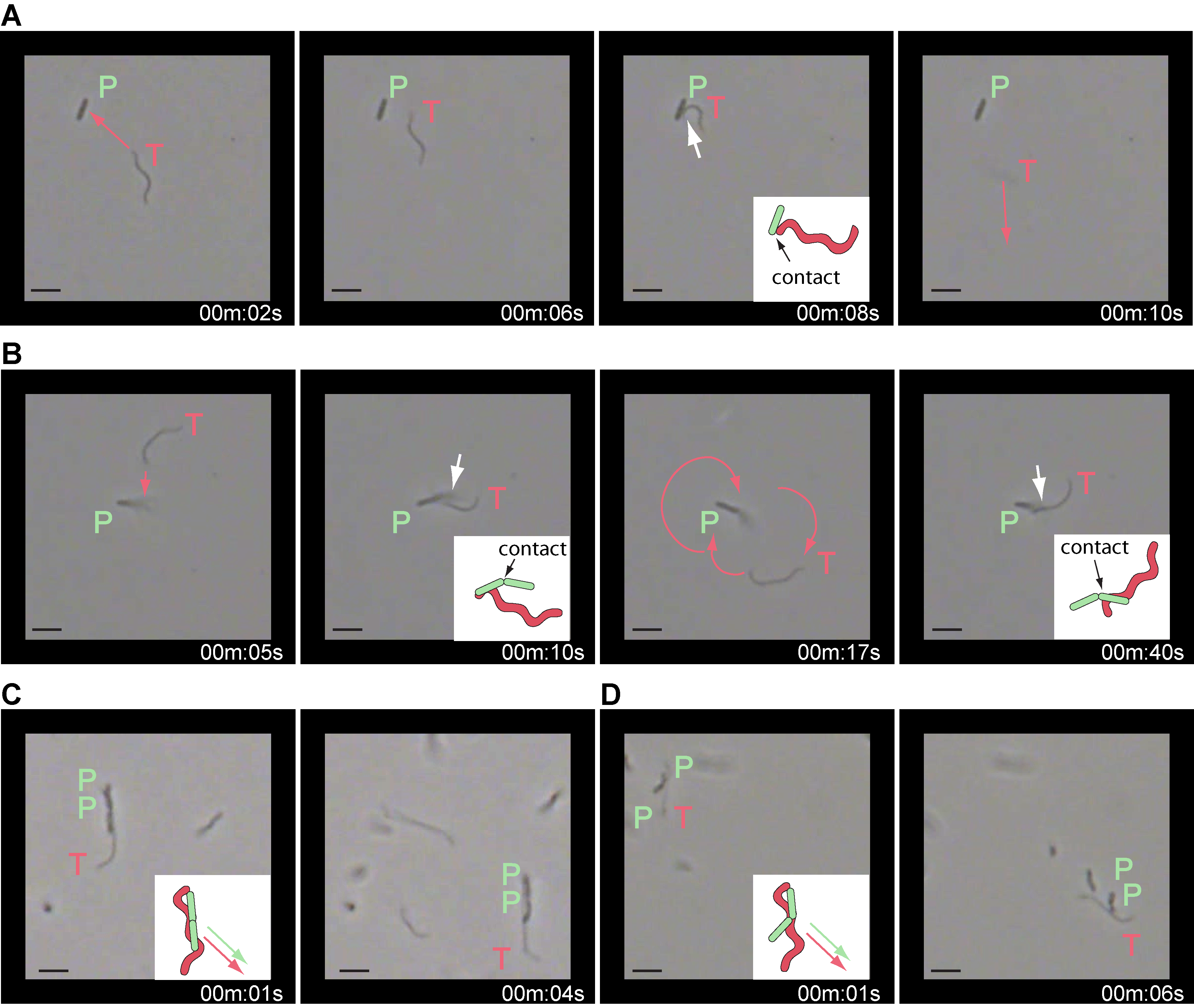

Supplement: S19 Fig — In the presence of Pseudomonas, T. parva demonstrates a behavioral shift and moves directly towards Pseudomonas and establishes a contact. (A) Frames selected from S3 Movie demonstrate position of one T. parva cell (T) and one Pseudomonas cell (P). Red arrow indicates direction of T. parva swimming, red arrow indicates the point of contact between T. parva and Pseudomonas. As seen on S3 Movie, T. parva detaches from Pseudomonas after a brief contact and swims in opposite direction (right panel). (B) Frames selected from S4 Movie demonstrate a recurrent contact between T. parva (T) and Pseudomonas (P) accompanied by a circular T. parva swimming around the Pseudomonas cell. (C) Frames selected from S5 Movie demonstrate a stable contact between one T. parva and two Pseudomonas cells resulting in a joint swimming of the bacteria. (D) Frames selected from S6 Movie demonstrate a stable contact between one T. parva and two Pseudomonas cells resulting in a joint swimming of the bacteria. (TIF) [file ppat.1008375.s019.tif]
